# Evolutionary information helps understand distinctive features of the angiotensin II receptors AT1 and AT2 in amniota

**DOI:** 10.1371/journal.pcbi.1009732

**Published:** 2022-02-24

**Authors:** Rym Ben Boubaker, Asma Tiss, Daniel Henrion, Hajer Guissouma, Marie Chabbert

**Affiliations:** 1 CNRS UMR 6015 – INSERM U1083, Laboratoire MITOVASC, Université d’Angers, Angers, France; 2 INSAT de Tunis, Université de Carthage, Carthage, Tunisie; Max Planck Institute for Biophysical Chemistry, GERMANY

## Abstract

In vertebrates, the octopeptide angiotensin II (AngII) is an important *in vivo* regulator of the cardiovascular system. It acts mainly through two G protein-coupled receptors, AT1 and AT2. To better understand distinctive features of these receptors, we carried out a phylogenetic analysis that revealed a mirror evolution of AT1 and AT2, each one split into two clades, separating fish from terrestrial receptors. It also revealed that hallmark mutations occurred at, or near, the sodium binding site in both AT1 and AT2. Electrostatics computations and molecular dynamics simulations support maintained sodium binding to human AT1 with slow ingress from the extracellular side and an electrostatic component of the binding free energy around -3kT, to be compared to around -2kT for human AT2 and the δ opioid receptor. Comparison of the sodium binding modes in wild type and mutated AT1 and AT2 from humans and eels indicates that the allosteric control by sodium in both AT1 and AT2 evolved during the transition from fish to amniota. The unusual S7.46N mutation in AT1 is mirrored by a L3.36M mutation in AT2. In the presence of sodium, the N7.46 pattern in amniota AT1 stabilizes the inward orientation of N3.35 in the apo receptor, which should contribute to efficient N3.35 driven biased signaling. The M3.36 pattern in amniota AT2 favours the outward orientation of N3.35 and the receptor promiscuity. Both mutations have physiological consequences for the regulation of the renin-angiotensin system.

## Author summary

The analysis of protein sequences from different species can reveal interesting trends in the structural and functional evolution of a protein family. Here, we analyze the evolution of two G protein-coupled receptors, AT1 and AT2, which bind the angiotensin II peptide and are important regulators of the cardiovascular system. We show that these receptors underwent a mirror evolution. Specific mutations at, or near, the sodium binding pocket occurred in both AT1 and AT2 during the transition to terrestrial life. We carried out electrostatics computations and molecular dynamics simulations to decipher the details of the sodium binding mode in eel and human receptors, as prototypes of fish and amniota receptors. Our results indicate that sodium binding is kinetically slow but thermodynamically stable. Comparison of the sodium binding modes in eel and human receptors reveals that an unusual mutation in the sodium binding pocket of AT1 is critical for biased signaling of amniota AT1 whereas a mutation in AT2 promotes promiscuity of amniota AT2. In turn, these data indicate that a few mutations at a strategic position (here the sodium binding pocket) are an efficient way to gain functional evolution.

## Introduction

The renin-angiotensin system (RAS) is an important *in vivo* regulator of multiple cardiovascular and renal functions in vertebrates [1–3]. It initiates with the cleavage of the angiotensinogen by renin into the decapeptide angiotensin I that is then cleaved by the angiotensin converting enzyme 1 (ACE1) into the octapeptide angiotensin II (AngII). AngII is subsequently cleaved into angiotensin 1–7 (Ang1-7) by the angiotensin converting enzyme 2 (ACE2). A variety of other angiotensin derivatives are produced by diverse enzymes. The RAS system is composed of two axes. In the “classical” axis, AngII binds the type 1 AngII receptor (AT1) to induce most known effects of AngII, including vasoconstriction and increased blood pressure, anti-natriuresis, hypertrophy, fibrosis and inflammation [4,5]. Dysregulation of this axis promotes cardiac and vascular damages and AT1 blockers are widely used to fight hypertension. The “counter-regulatory” axis involves the type 2 AngII receptor (AT2) and the MAS receptor, through AngII (AT2) and Ang1-7 (AT2, MAS). These receptors promote vasodilation, natriuresis, anti-inflammation, anti-fibrosis, and anti-proliferative responses that counterbalance AT1 effects [3,6,7].

AT1 and AT2 are two G protein-coupled receptors (GPCRs) from class A that share 30% sequence identity and similar affinity for AngII but have very different properties, expression profiles and functions [4]. AT1 is expressed in all organs. When it is stimulated by AngII, it activates both G proteins and β-arrestins. A variety of AT1 ligands induce biased signaling with preferential activation of G proteins or β-arrestins [8,9]. AT2 is widely expressed during development whereas, in the adult life, it is expressed only in a few organs, except in pathological conditions where its expression is up-regulated [5,10,11]. Direct AT2 activation of the conventional GPCR effectors (G proteins and β-arrestins) remains controversial and the primary signaling pathways involved in AT2 signaling are unknown [12–14]. A better knowledge of these pathways is important as stimulation of AT2 in pathological situations might be an interesting alternative to the blockade of AT1 [10].

Several structures of AT1 [15–18] and AT2 [19–21] in complex with different ligands have been resolved. AT1 has been crystallized in both inactive and active states. AT2 has been crystallized in active states only, which supports the hypothesis of a “relaxed” conformation that would be prone to activation [22]. Comparison of the active structures of AT1 in complex with AngII and biased agonists provides clues to the biased responses of AT1 [16] that match those observed in large-scale molecular dynamics simulations [23]. Briefly, two conformations of the active state are differentially stabilized by β-arrestin-biased, balanced, and G protein-biased ligands. Two asparagine residues located at positions 3.35 and 7.46 (Ballesteros’ numbering [24]) play a key role in this mechanism. The inward/outward orientation of N3.35 acts as an allosteric switch between the balanced canonical and the β-arrestin-biased alternative conformations, whereas reorganization of the N7.46 H-bonding pattern helps transmit the information to the intracellular side [16,23]. These two residues are located at an allosteric sodium binding site that is highly conserved among class A GPCRs.

Sodium binding is an essential element of receptor activation in most class A GPCRs. It acts as a negative allosteric regulator, by stabilizing the inactive structure, and might be involved in the activation mechanism [25,26]. Sodium coordination involves a strictly conserved aspartic acid at position 2.50 and polar residues at positions 3.35, 3.39, 7.45, 7.46, or 7.49. An asparagine at position 3.35 is frequent in GPCRs, whereas an asparagine at position 7.46 is unusual [25,27]. In the inactive structures of human AT1, N7.46 is H-bonded to N3.35 [17,18]. It was suggested that the N7.46 pattern might prevent sodium binding while stabilizing the inactive structure by interacting with N3.35 [16]. By contrast, human AT2 has a conventional sodium binding site, with the N3.35 and S7.46 patterns.

Since residues from the sodium pocket have a functionally important role in AT1 activation, a better knowledge of the history of this site is mandatory to understand the role of this unusual sodium pocket, not only in the activation mechanism of AT1, but also in the balance between AT1 and AT2 activation. This prompted us to analyze the evolutionary information contained in the extend sequences of both AT1 and AT2.

The evolutionary approach revealed a mirror evolution of AT1 and AT2. Both AT1 and AT2 are split in two clades that separate fish from terrestrial receptors. Mutations of residues at, or close to, the sodium binding site occurred specifically in amniota receptors. In particular, the N7.46 pattern in AT1 is an innovation of amniota. To understand the effects of the observed mutations, we carried out molecular dynamics (MD) simulations and electrostatics computations to determine (1) the sodium ingress pathway to the canonical binding site in AT1 and (2) the characteristics of the sodium binding mode in AT1 and AT2 receptors from humans and eels. The mutations specific of amniota AT1 and AT2 receptors have physiological consequences for the regulation of the renin-angiotensin system that will be discussed.

## Results

### Molecular evolution of the angiotensin II receptors

**Fig 1:**
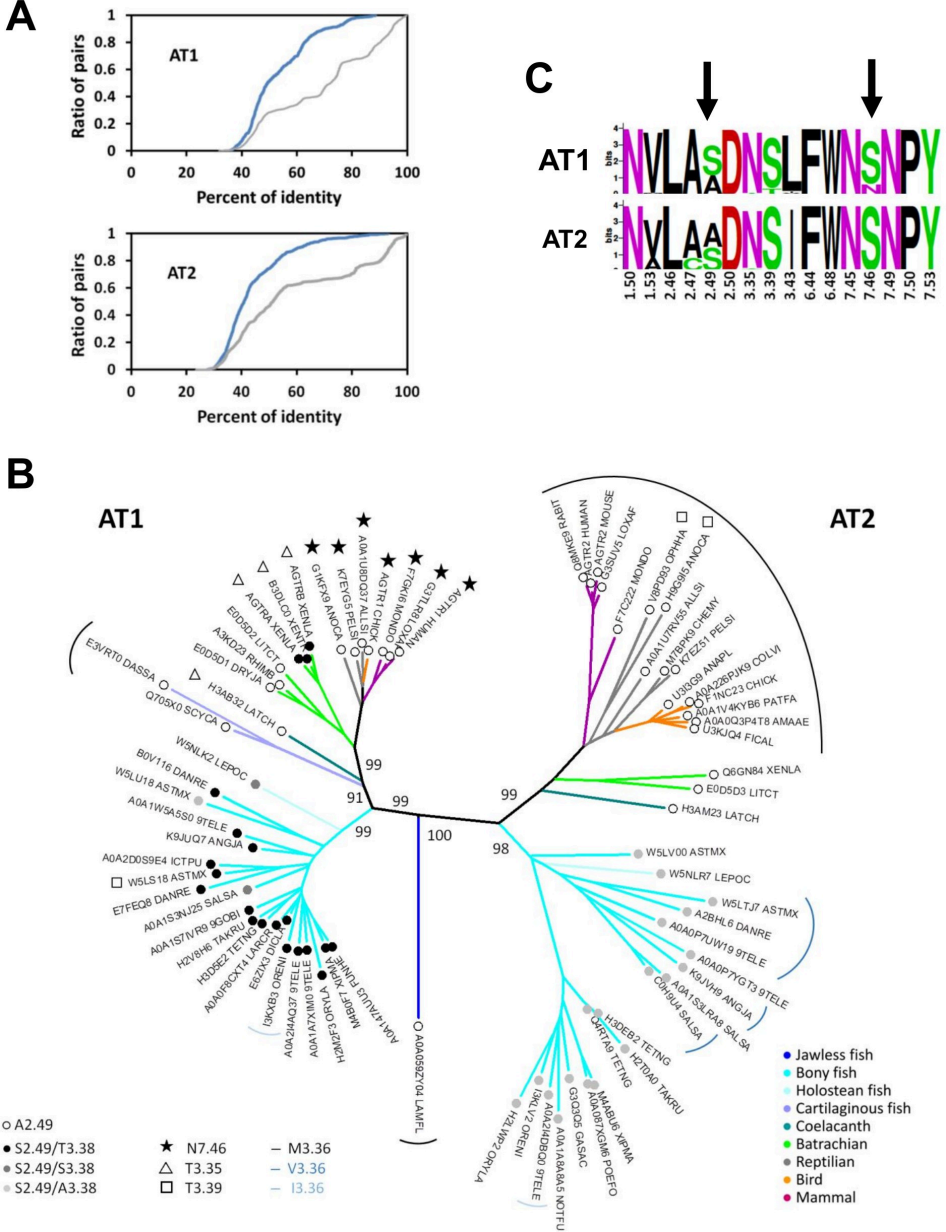
Evolution of the angiotensin II receptors. (A) Cumulative distribution function of the pairwise identities for AT1 (top) and AT2 (bottom), in the Uniprot sets (grey lines) and in the non-redundant sets (blue lines); (B) Neighbor-joining tree of the non-redundant set of AT1 and AT2 receptors (500 bootstraps). The boostrap values in percent are indicated for main branches. The color code of the tree branches refers to the phylum (jawless fish: dark blue, bony fish: cyan, holostean fish: light cyan, cartilaginous fish: slate, coelacanth: deepteal, batrachian: green, reptilian: grey, bird: orange, mammal: purple). The external labels indicate mutations in the sodium binding site as compared to the canonical sodium binding site characterized by N3.35, S3.39 and S7.46 (N7.46: stars, T3.35: open triangles, T3.39: open squares). The internal labels indicate the residues at positions 2.49 and 3.38 (A2.49: open circle, S2.49/T3.38: black circle, S2.49/S3.38: dark grey circle, S2.49/A3.38: light grey circle). The circular arcs indicate the residue at position 3.36 (black: M3.36, dark blue: V3.36, light blue: I3.36, no arc: L3.36); (C) Sequence logo plots of the sodium binding site in the NR set of AT1 and AT2. For these plots, all the residues lining up the sodium binding site were taken into account [23]. The arrows highlight the mutations at positions 2.49 and 7.46.

Data mining from Uniprot (see Methods) provided a set of 318 “clean” sequences of AngII receptors. Based on a preliminary tree, the sequences were assigned to either the AT1 or AT2 sub-family (203 and 115 members, respectively). These receptors presented strong sequence biases. Only 28% of AT1 pairs and 51% of AT2 pairs had a sequence identity lower than 50% (Fig 1A). To obtain a non-redundant set of vertebrate receptors, we clustered the 318 sequences using the NRDB program [28], with a threshold of 90% sequence identity. We obtained a total of 75 clusters: 37 clusters for the AT1 sequences and 38 clusters for the AT2 sequences. The 120 AT1 and 68 AT2 sequences from mammalians clustered into 3 and 5 groups, respectively. The clusters containing human AT1 and AT2 included 115 and 39 sequences, respectively.

We selected a representative sequence from each cluster, based on sequence and genome qualities, to obtain a non-redundant (NR) sequence set. In this set, 51% and 74% of, respectively, AT1 and AT2 pairs had less than 50% identity (Fig 1A). Using the aligned sequences of the NR set, we built a Neighbour Joining phylogenic tree of the AngII receptors. Starting from a single AngII receptor more closely related to AT1 in jawless fish (lamprey), duplication in an ancestor of jawed fishes led to an almost symmetrical evolution of the AT1 and AT2 receptors, hinting at their complementary role in RAS control (Fig 1B). Each AT1 and AT2 sub-tree possesses two major clades: one for fishes and one for terrestrial animals. There are two exceptions to this pattern: (1) The coelacanth AT1 and AT2 sequences are clearly part of the “terrestrial” clades, in the vicinity of the batracian sequences. Their position is consistent with the coelacanth status of “living fossil”, in an intermediary position in the tree of life; (2) Only AT1 sequences are present in available cartilaginous fish genomes (rays, sharks), as detailed in Methods. These sequences are closer to terrestrial than to fish sequences. Interestingly, this feature was observed for the full genome of sharks [29].

Human AT1 possesses an unusual sequence pattern (N7.46) at the canonical sodium binding site. To determine when this pattern emerged, we analyzed the residues lining the sodium binding site [25,26]. After verifying that all the sequences from each cluster presented the same pattern, we reported key positions on the phylogenetic tree (Fig 1B). Most residues are highly conserved between and within the AT1 and AT2 sets (Fig 1C). In particular, residues usually involved in sodium coordination (D2.50, N3.35, S3.39 and N7.45) are highly conserved. A difference is observed at position 3.43 with Leu and Ile for AT1 and AT2, respectively. In both sets, the position 2.49 differentiates fish (S2.49) from terrestrial (A2.49) receptors (Fig 1B and 1C). The C2.47 pattern is present only in some AT2 sequences from fishes. Noteworthy, as observed in most GPCRs, a serine residue at position 7.46 is present in most AT1 and AT2 receptors (Fig 1B). An Asn residue at this position is observed only in amniota (reptilians, birds, and mammals). This indicates that this specificity of AT1 has been acquired during the transition from an aqueous to a terrestrial environment.

A “canonical” sodium binding site with two Asn residues at positions 3.35 and 7.45 and two Ser residues at positions 3.39 and 7.46 is thus observed in AT2 receptors from any species and in AT1 receptors from fishes and some amphibians (Neobatracia clade). The N7.46 pattern is observed specifically in amniota whereas other coordinating residues are conserved. Notably, a N3.35T mutation of the residue facing S7.46 is observed in the coelacanth and batracians from the pipoidea clade (*Xenopus*), suggesting that evolution might have led to different solutions for a similar problem.

### Properties of D2.50 in AT1 and AT2

**Fig 2:**
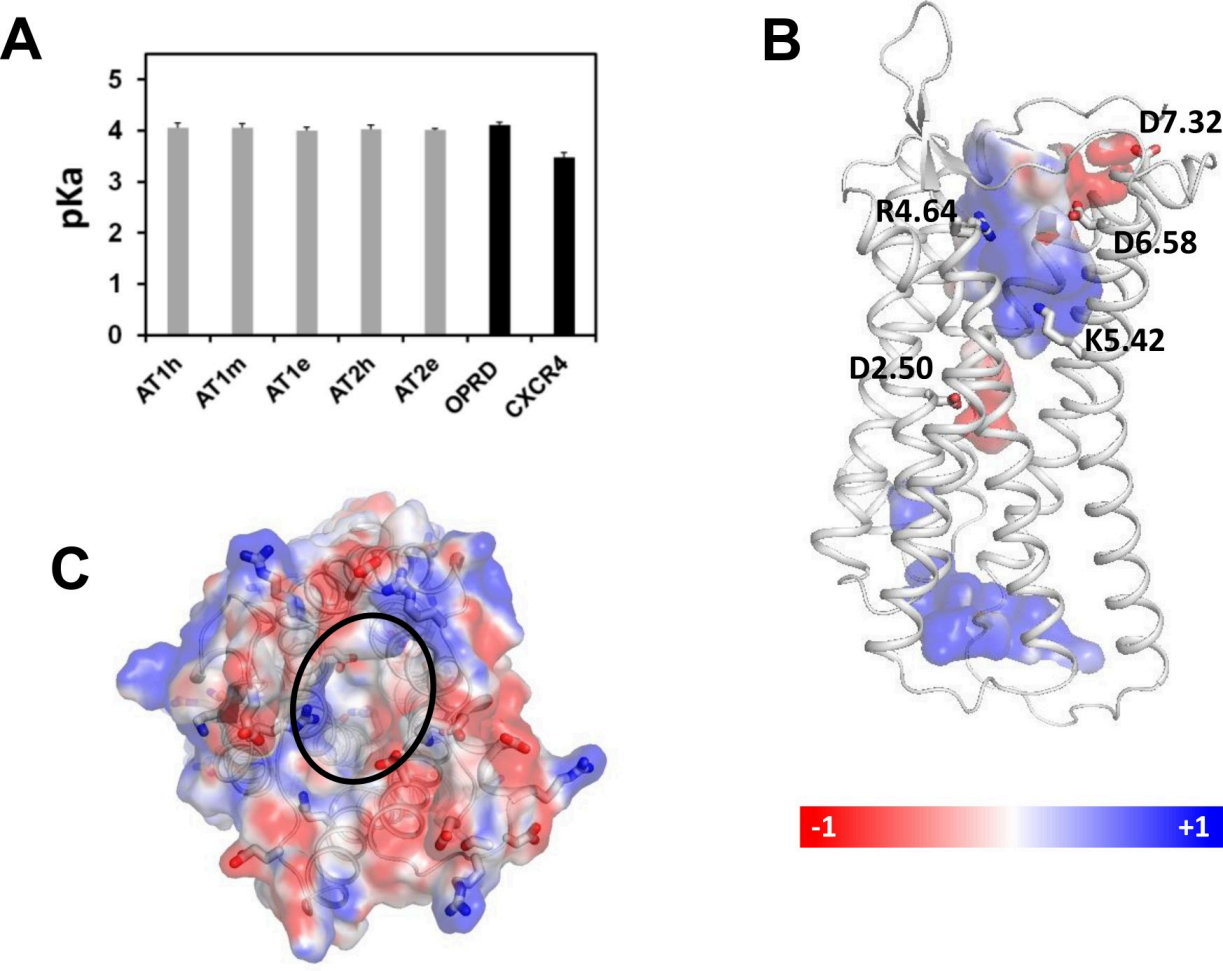
Electrostatic properties of AT1 and AT2. (A) pKas of residue D2.50 in AT1 and AT2 receptors and in the AT1m variant. The pKas of D2.50 in CXCR4 and OPRD are given for comparative purpose; (B) Electrostatic potential of the AT1h internal cavities (side view). The conserved charged side chains interacting with AngII (R4.64, K5.42, D6.58 and D7.32) and D2.50 are shown as sticks; (C) Electrostatic potential of the extracellular surface of AT1h (top view). The surfaces are colored from red (-1eV) to blue (+1eV).

The deprotonation of D2.50 is a key element of sodium binding to GPCRs. We thus wondered whether the mutations in the sodium binding site of AT1 and AT2 could alter the pKa of D2.50. To answer this question, we selected AT1 and AT2 from *Anguilla japonica* (eel) as prototypes of the fish receptors. The receptors were modeled from the crystallographic structures of human AT1 and AT2 (see Methods). We computed the pKa of D2.50 in AT1 and AT2 from humans (AT1h, AT2h) and eels (AT1e, AT2e) and in the human AT1 variant with the N7.46S mutation (AT1m). The pKa of D2.50 in the δ opioid receptor (OPRD) and in the chemokine receptor CXCR4 were also computed as controls. To calculate pKas, we used the DelphiPKa program with the model of Gaussian representation of atomic densities proposed by Alexov and coworkers [30–32]. This representation yields a smooth Gaussian-based dielectric function without defining a molecular surface. Electrostatic computations depend on two parameters: the width of the Gaussian function, σ, and the protein dielectric value, εin. We compared a wide range of parameters (σ varying from 0.5 to 1, εin varying from 2 to 10) for AT1h and OPRD in search of pairs compatible with sodium binding to OPRD (see Methods). Each pair of parameters led to similar pKa values for AT1 and OPRD (S1 Fig), indicating that D2.50 has the same protonation state in both receptors. Noteworthy, sodium binding to OPRD is not compatible with the default values for internal groups in a hydrophobic environment (σ = 0.9, εin = 4), which lead to neutral pKa (7.3 ± 1.0), but supports the default values for external groups at protein surface (σ = 0.7, εin = 8), which lead to a pKa of 4.1 ± 0. These latter parameters are consistent with the location of D2.50 facing a water filled cavity. Using these parameters, we obtained similar values for the pKa of D2.50 in AT1 and AT2 from both humans and eels and in the AT1m variant (Fig 2A). This indicates that the presence of N7.46 in the sodium binding site does not alter the physico-chemical properties of D2.50 in human AT1. Notably, we observed a significant decrease in the pKa of D2.50 in CXCR4 due to the presence of a histidine at position 7.45. As a consequence of the pKa of about 4 for D2.50 in AT1h, the sodium pocket has a negative electrostatic potential (Fig 2B) and should bind a sodium ion in case this one reaches the allosteric site.

### Sodium ingress pathway

These findings prompted us to initiate a kinetics study to determine whether the sodium ion could reach the allosteric site. We modeled AT1h without sodium and embedded the receptor into a hydrated POPC bilayer, in the presence of NaCl, both on the extracellular and intracellular water layers. These conditions allow testing the possibility of ingress from either the extracellular or the intracellular side, since both pathways have been observed [33]. We used CXCR4 and OPRD as controls. We did observe fast sodium binding, from the extracellular side, within 10 and 100 ns of classical MD simulations for, respectively, CXCR4 and OPRD, in the presence of 0.15M NaCl (S2 Fig). These data are consistent with previous observations by others [33]. Binding of a sodium ion to AT1h was not as easy and the same conditions (0.15M NaCl, classical MD simulations) failed for AT1h on the sub-microsecond timescale. Since our aim was the feasibility of sodium binding and not the details of the sodium entrance that might necessitate tens of microseconds [33], we used “harsh” conditions, by combining high sodium concentration (1M NaCl) and accelerated MD simulations (see Methods), in an attempt to speed up the kinetics.

**Fig 3:**
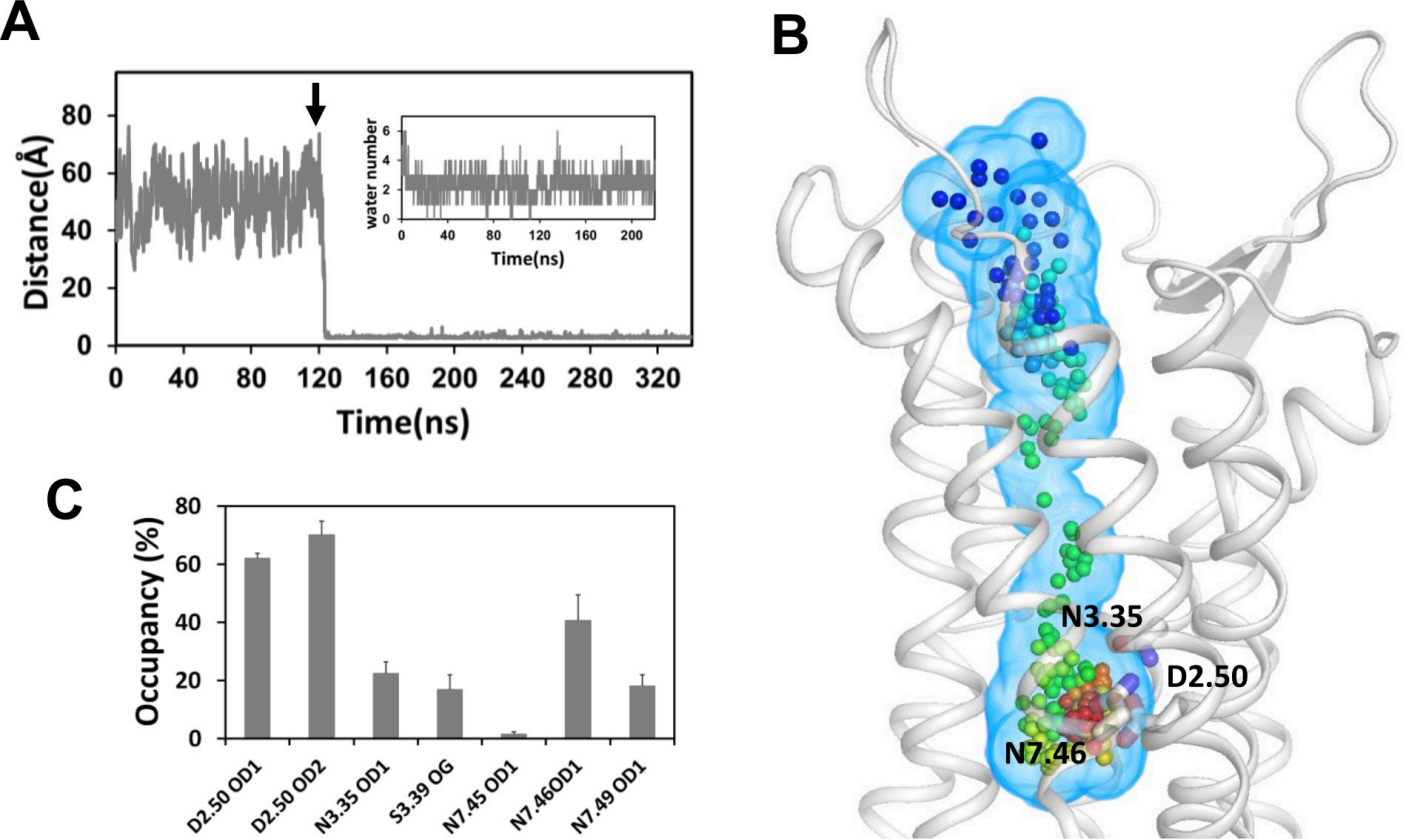
Kinetics of sodium binding to AT1h. (A) Trajectory of the sodium ion reaching the canonical binding site in AT1h, measured by the distance between the sodium ion and the CG atom of D2.50. The arrow indicates the beginning of the accelerated MD simulations after 120 ns of classical MD simulation. The insert represents the number of water molecules in the first coordination shell of the sodium ion from the beginning of the acceleration; (B) Equidistant snapshots of the sodium pathway to the canonical sodium binding site, superposed on the ribbon representation of AT1h (white). The snapshots were extracted every 8 ps, from 1.7 ns to 4.8 ns after the beginning of the acceleration. The rainbow color from blue to red indicates the time. The volumetric map in light blue indicates the volume occupied by the ion during its pathway to the allosteric site. Side chains of D2.50, N3.35 and N7.46 are shown as sticks; (C) Coordination of the sodium ion with protein atoms after desolvation (data are indicated as average ± standard deviations of three slots in the 20–220 ns range of the aMD trajectory).

After a 120 ns long classical MD step, we initiated acceleration. Under these conditions, we could observe the ingress of a sodium ion into the receptor internal cavity, and its pathway towards the allosteric pocket (Fig 3A and 3B). This step was completed in less than 10 ns of accelerated simulations and was followed by the desolvation of the ion from 5–6 to 2–3 water molecules in the first shell. After desolvation, the sodium ion was coordinated mainly to the D2.50 OD1 and OD2 atoms and to the N7.46 OD1 atom (Fig 3C), as observed under steady-state conditions (see below).

To understand the slow kinetics of sodium ingress, we analyzed the electrostatic potential of the receptor. The extracellular surface of AT1h displayed a complex electrostatic pattern whereas the orthosteric internal cavity was highly dipolar (Fig 2B and 2C). This polarity is due to strictly conserved positive (K5.42 and R4.64) and negative (D6.58 and D7.32) residues that interact with, respectively, the negative carboxyl terminal group and the positive Arg2 side chain of AngII in both AT1 [16] and AT2 [19]. Consequently, the sodium ion ingress is hindered by the strong electrostatic barrier due to K5.42 and R4.64, but remains possible. Similar electrostatic potentials (S3 Fig) were observed in AT1 and AT2 from both eels and humans, and in the AT1m variant for which sodium binding has been evidenced experimentally [34]. Noteworthy, the fast sodium ingress in CXCR4 and OPRD correlates with the negative potential of their extracellular cavity (S3 Fig).

### Sodium binding mode in the human AT1 and AT2 receptors

**Fig 4:**
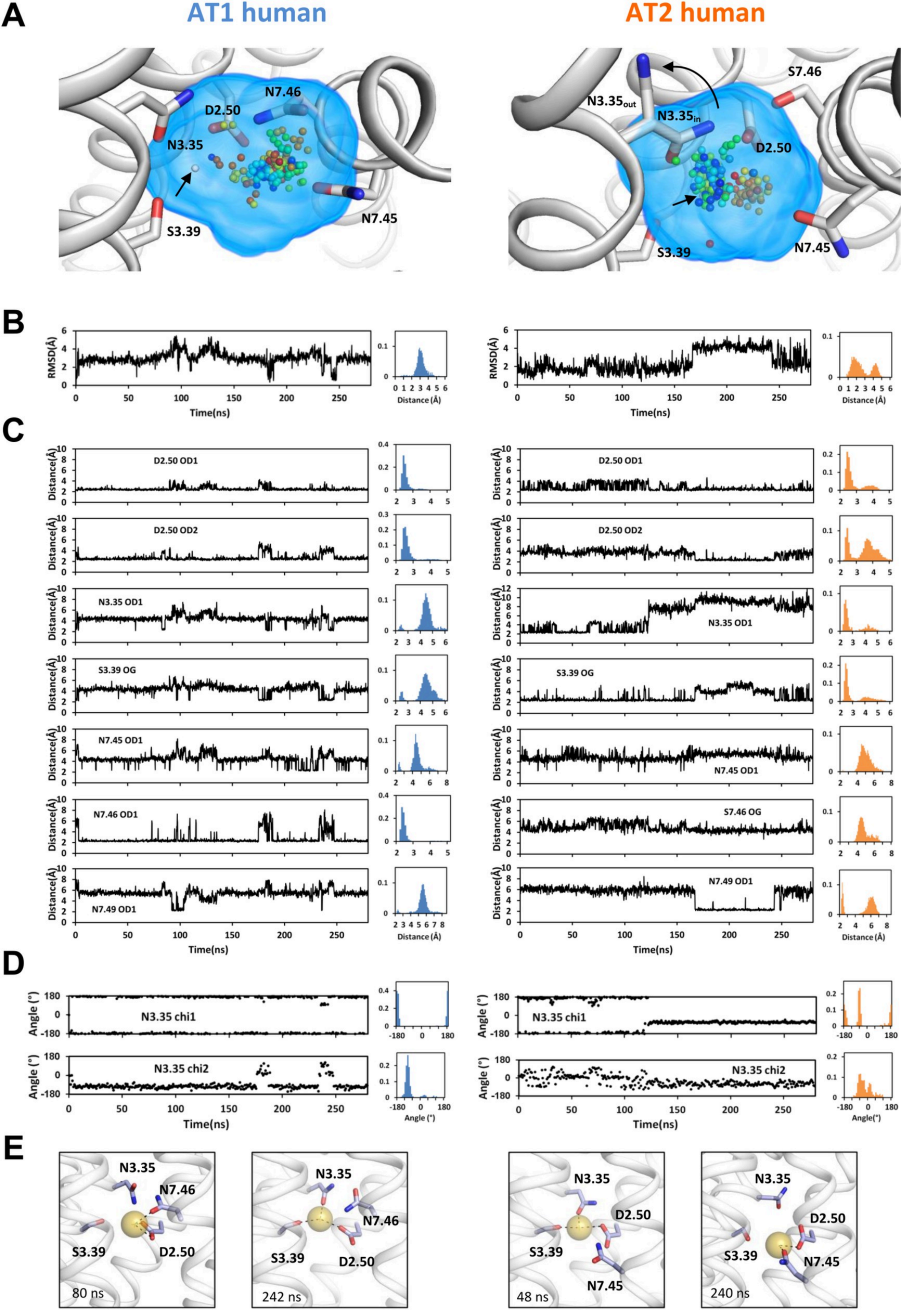
**Sodium coordination in representative trajectories of AT1h (left) and AT2h (right).** (A) Positioning of the sodium ion as a function of time (from blue to red), during a 280 ns long trajectory, superposed on the receptor initial conformation (white ribbon and sticks). The initial position of the sodium ion is indicated by a white sphere and an arrow. For AT2h, N3.35 is shown in both the initial inward and final outward orientations; (B) Root-mean square deviations (RMSD) of the sodium ion, and histograms of the deviations; (C) Distances of the sodium ion to the putative protein ligands and corresponding histograms; (D) Dihedral angles of N3.35 and corresponding histograms; (E) Snapshots showing how side chain rotamerization affects sodium coordination. For AT1, the left snapshot (80 ns) shows interaction of the sodium ion with D2.50 and N7.46. N3.35 is in the *trans* chi1 and *g-* chi2 conformation. The right snapshot (242 ns) shows the interaction of the sodium ion with N3.35 upon the transient rotamerization of the N3.35 chi2 angle. For AT2, the left snapshot (48 ns) shows N3.35 in the inward, *trans* orientation and the sodium ion interacting with D2.50, N3.35, and S3.39. The right snapshot (240 ns) shows N3.35 in the outward, *g-* orientation and the sodium ion interacting with D2.50 and N7.45.

We carried out MD simulations of the human AT1 and AT2 receptors modeled in the inactive state, with a sodium ion located at the canonical site. In both models, N3.35 was initially oriented inward, facing the sodium site, in the *trans* orientation, as observed in the inactive structures of AT1 [17,18]. In Fig 4, we show typical trajectories and analyses for AT1h and AT2h. We monitored sodium motion within its binding site, the RMSD of the ion from its initial position, the distances to the putative protein ligands, the orientation of N3.35, and corresponding histograms (Fig 4A–4D). Based on distance distributions, the threshold for interaction was set at 2.8 Å.

In AT1h (Fig 4, left panels), the initial position of the sodium ion near N3.35 was not stable and the sodium rapidly moved by about 3 Å towards a position in the vicinity of D2.50 and N7.46. In this position, the sodium ion interacted with the OD1 and OD2 atoms of D2.50 and the OD1 atom of N7.46. From this position, the sodium ion transitorily escaped to interact with N3.35, N3.39, N7.45, or N7.49. These interactions were similar to those observed for the sodium ion that reached the allosteric site from the extracellular compartment (Fig 3C). The interactions depended on both the sodium position and the dihedral orientation of the side chains. As examples, the snapshots in Fig 4E show that transient interactions of the ion with N3.35 occurred only when the chi1 and chi2 dihedral angles of N3.35 were, respectively, in the *trans* and *g+* conformations.

**Fig 5:**
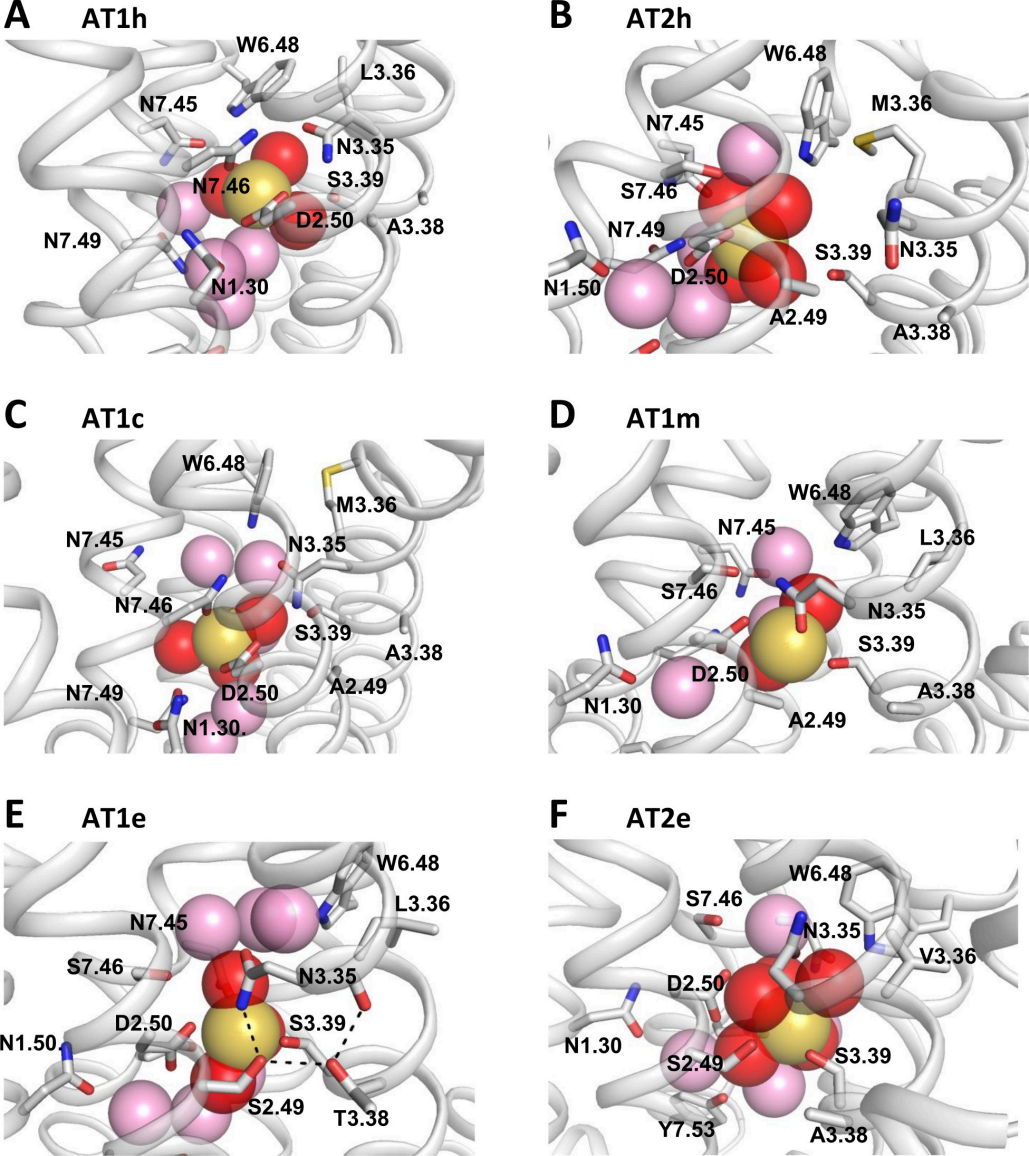
Snapshots of the sodium binding site in AT1 and AT2. (A) In the AT1h snapshot, the sodium ion is coordinated to the D2.50 OD1 and OD2 atoms, to the N7.46 OD1 atom, and to three water molecules; (B) In the AT2h snapshot, the sodium ion is coordinated to the D2.50 OD1 atom and to four water molecules. N3.35 is in the outward orientation; (C) In the AT1c snapshot, the sodium ion is coordinated to the D2.50 OD2 and N7.46 OD1 atoms, and to three water molecules; (D) In the AT1m snapshot, the sodium ion is coordinated to the D2.50 OD1, N3.35 OD1, and S3.39 OG atoms, and to two water molecules; (E) In the AT1e snapshot, the sodium ion is coordinated to the D2.50 OD2 and S3.39 OG atoms, and to three water molecules. The dash lines indicate the H-bonds linking the N3.35 ND2, S3.39 OG, T3.38 OG1, and F3.31 O atoms; (F) In the AT2e snapshot, the sodium ion is coordinated to the S3.39 OG atom and to five water molecules. N3.35 is in the *g+* orientation (chi1 = 87°). D2.50 is in the second shell. In (A-F), the sodium ion is shown as a yellow sphere. Water molecules participating to the first coordination shell are shown as red spheres. Other water molecules within 6 Å from the sodium ion are shown as pink spheres.

In addition to interacting with protein atoms, the sodium ion also interacted with neighbour water molecules to complete the first coordination shell. These water molecules bridged the sodium ion to either electronegative atoms of the sodium cavity or to other water molecules to form a second coordination shell. A typical snapshot of sodium coordination in AT1h is shown in Fig 5A. The sodium ion directly interacted with the D2.50 OD1, D2.50 OD2, and N7.46 OD1 atoms and with three water molecules. N3.35 did not interact directly with the sodium ion, but through a water molecule, thus contributing to the second coordination shell.

The sodium trajectory was markedly different in AT2h (Fig 4, right panels). Initially, the sodium ion remained near the modeling position (1–2 Å) where it interacted with the D2.50 OD1, N3.35 OD1, and S3.39 OG atoms. However, after about 120 ns, N3.35 underwent an outward rotamerization, modifying the sodium binding cavity (Fig 4A). The sodium moved up to 4–5 Å from the initial position, first towards N7.49 (coordination with N7.49 and D2.50) and then back towards S3.39 in a position where it was coordinated mainly to the D2.50 OD1 atom and, intermittently, to the S3.39 OG atom. Sodium coordination was completed by water. The snapshot in Fig 5B, extracted after the outward rotamerization of N3.35, shows the sodium ion coordinated to the D2.50 OD1 atom and four water molecules.

### Evolution of the sodium binding mode

**Fig 6:**
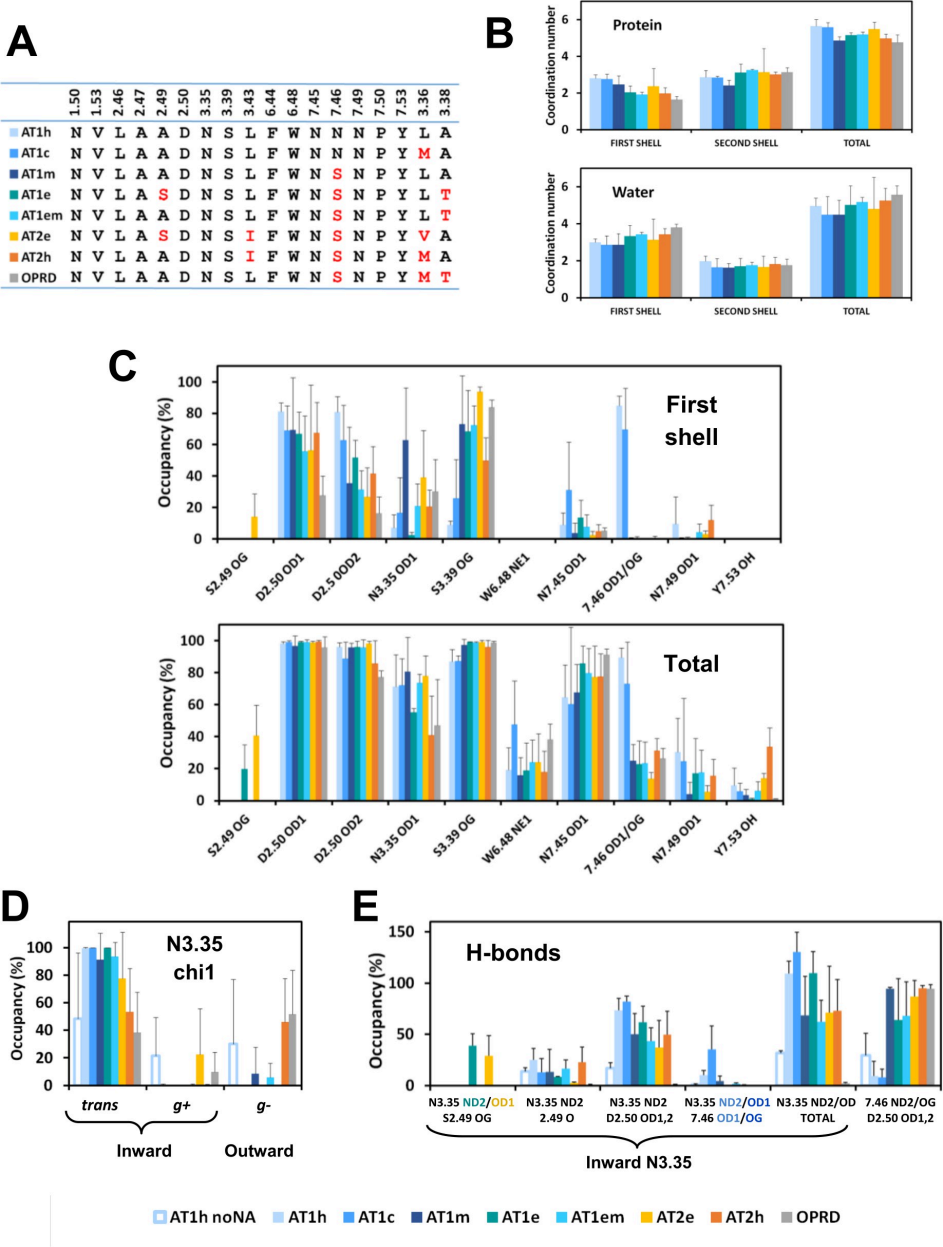
Coordination of the sodium ion in AT1 and AT2 receptors. (A) Residues lining the sodium binding cavity and neighbour positions 3.36 and 3.38 in the receptors under scrutiny; (B) Number of coordination of the sodium ion to protein ligands (top) and water molecules (bottom) in the first shell, in the second shell, and in either shell; (C) Average occupancy of the protein ligands in the first shell (top) and in either the first or the second shell (bottom); (D) Distribution of the chi1 dihedral angle of N3.35 observed during the simulations; (E) Occupancy of the H-bonds stabilizing the sodium binding site. For N3.35 H-bonds, only frames with inward N3.35 were taken into account. In (B-E), the error bars represent standard error of the mean among replica. The receptors are indicated by the following color code: AT1h: light blue, AT1c: middle blue, AT1m: dark blue, AT1e: deepteal, AT1em: cyan; AT2e: yellow, AT1h: orange, OPRD: grey.

To decipher the consequences of the mutations observed in the sodium binding site during the evolution of the AT1 and AT2 receptors, we analyzed the coordination of the sodium ion in a variety of AT1 and AT2 receptors. In addition to human receptors, we investigated AT1 and AT2 from eels, as prototypes of fish receptors, three variants and OPRD. The N7.46S mutation in AT1h (AT1m) reversed the evolutionary observed S7.46N mutation. In the chimeric AT1h variant (AT1c), a fragment of transmembrane helix 3 (TM3), from position 3.25 to 3.40, was replaced by the equivalent sequence of AT2h. The AT1c variant has a constitutive activity not observed for AT1 [35]. The S2.49A AT1e variant aimed at analyzing the importance of S2.49 in the sodium binding mode of eel receptors. Finally, OPRD was chosen for comparative purposes because its sodium cavity is similar to AT1 and AT2 and its sodium binding mode has been studied [26,36]. The residues lining the sodium cavity of these receptors are reported in Fig 6A.

As previously, in all the initial models, the sodium ion was positioned near D2.50 and N3.35, as observed in the crystal structure of OPRD, and N3.35 was oriented inward. All the simulations were replicated at least 3 times starting from the same construct. AT1h and AT2h were replicated 6 times from two constructs (see Methods). In all the simulations, the receptors were stable during the trajectory (S4 Fig). The RMSD deviations of the TM helices increased up to around 2 Å at the beginning of the simulations and then reached a plateau after 20–40 ns of simulations. High RMSF were observed for loops whereas TM helices were stable, especially on the intracellular side. The distance between TM3 and TM6, which is the main marker of receptor activation, ranged between 6 and 8 Å, indicating stable inactive structures.

The RMSD of the sodium ion revealed differences, both within and between simulations sets (S5 Fig). Highest RMSD were observed for AT1h and AT1c (3.2 ± 0.9 Å and 2.8 ± 1.1 Å, respectively), the two receptors with an Asn residue at position 7.46. For AT2h, the RMSD increased after outward motion of N3.35 as observed in Fig 4 (2.6 ± 1.1 Å). The RMSDs of the sodium ion in the other receptors were ≤ 2 ± 1 Å. In individual runs, the sodium position fluctuated by about 1Å, with at least partial overlap between runs of the same receptor (S5 Fig). The only exception was observed for AT2e with two narrow sub-sites (RMSD around 0.5 Å) that did not interconvert during the simulations.

Analysis of sodium coordination was based on the distances between the sodium ion, electronegative protein groups and water oxygens, to determine residues and water molecules contributing to the first and second coordination shells (see Methods). Heatmaps of the distances between the sodium ion and putative ligands are given for each individual trajectory as S6 Fig. Because the sodium environment is dynamical with switches of neighbour groups or water molecules between the first and second shells, we also considered both shells together (“total” shell). The results are summarized in Fig 6B and 6C.

The average number of coordination to protein ligands in the first shell decreased from 2.8 ± 0.2 for AT1h and AT1c to 2.0 ± 0.3 for AT1e and AT2h, to be compared to 1.6 ± 0.2 for OPRD (Fig 6B). The first shell was completed by two to five water molecules. This variability is exemplified in Fig 5 where the number of water molecules in the first shell is 2 for AT1m, 3 for AT1h, AT1e, and AT1c, 4 for AT2h, and 5 for AT2e. The average number of water molecules in the first shell varied from 3.0 ± 0.2 for AT1h to 3.4 ± 0.3 for AT2h and 3.8 ± 0.2 for OPRD. The second shell included around three protein ligands and two water molecules without significant differences between the receptors under scrutiny (Fig 6B). When both shells were taken into consideration, the total number of coordination to protein groups ranged from 5.7 ± 0.4 for AT1h to 5.0 ± 0.2 for AT2h and 4.8 ± 0.4 for OPRD. In any case, four to six water molecules were included in either the first or the second shell, indicating that the presence of an Asn residue at position 7.46 does not significantly reduce the size of the allosteric site or the number of water molecules in the environment of D2.50.

The occupancy rate of the putative ligands is reported in Fig 6C and highlights the specificities of the sodium binding mode in each receptor. Representative snapshots illustrating the variety of sodium coordination are reported in Fig 5.

Both in the AT1h receptor and in the AT1c variant, the sodium ion interacted preferentially with D2.50 (two coordinations) and N7.46 (Fig 6C). In both cases, N3.35 remained in the *trans* orientation (S7 Fig) and, along with S3.39, it participated in the second coordination shell (Fig 6C). A different binding mode was observed for the AT1m variant. In this case, the sodium ion interacted preferentially with the D2.50 OD1, N3.35 OD1, and S3.39 OG atoms. This is similar to the binding mode in the AT2h receptor with inward orientation of N3.35 (Fig 4) and corresponds to the binding mode observed in the crystal structure of OPRD [36].

The AT1e receptor has the same S7.46 pattern as the N7.46S variant but presented a different binding mode, with no coordination to N3.35 in the first shell. In this receptor, a network of polar interactions involving N3.35, D2.50, S2.49, and T3.38 stabilized the position of the N3.35 OD1 atom at about 4 Å of the sodium ion (Fig 5E). Thus, N3.35 did not participate in the first coordination shell but was part of the second shell. In the AT1em variant with an alanine at position 2.49, direct coordination of the sodium ion to N3.35 was restored (Fig 6C).

The AT2e receptor presented unique features among receptors with two narrow sub-sites (S5 Fig). In sub-site 1 (replicas 1 and 3), the sodium interacted preferentially with the D2.50 OD1, N3.35 OD1, and S3.39 OG atoms (occupancy of 80 ± 5%, 56 ± 1%, and 95 ± 4%, respectively). Additional coordination to the S2.49 OG or the D2.50 OD2 atom was also possible. Sub-site 2 (replica 2) was highly unusual with N3.35 in the *g+* orientation, pointing towards W6.48 (Fig 5F). The sodium ion interacted directly with S3.39 only. It also interacted through water molecules with the S2.49 OG, D2.50 OD1 and OD2, N3.35 OD1, W6.48 NE1, and N7.45 OD1 atoms (occupancy of 60, 89, 86, 87, 44, and 54%, respectively).

In OPRD, the interaction of the sodium ion observed in the sodium structure with N3.35 [36] was lost during the MD simulations, due to N3.35 rotamerization, as observed by Filizola et co-workers [37]. In particular, the outward rotation of N3.35 in OPRD was observed in the three runs that have been carried out (S7 Fig). This behaviour was similar to AT2h for which the outward rotation of N3.35 was observed in five out of the six AT2h replicas (S7 Fig). Sodium binding in OPRD was dominated by interactions with S3.39 and, to a lesser extent, with D2.50.

### Stability of the sodium binding site through H-bonds

The H-bonds between the polar residues of the sodium binding site contribute to the stability of the receptor. With this regard, comparison of the H-bonding pattern of human AT1 in the presence and absence of sodium is essential to understand the role of the sodium ion in AT1 regulation. Thus, we carried out MD simulations of AT1h in the absence of sodium to determine whether the H-bonding pattern observed between N3.35 and N7.46 in the crystal structures of inactive AT1 [17,18] could stabilize the structure of the sodium-free apo receptor, under dynamical conditions.

We compared the H-bonds involving positions 3.35 and 7.46 in the receptors under scrutiny. As previously pointed out, N3.35 can undergo an outward rotamerization. In this latter orientation, it cannot interact with residues from the sodium binding site (Fig 5B). Thus we monitored the chi1 angle of N3.35 in all the simulations (S7 Fig) and analyzed the N3.35 H-bonding pattern in the inward orientation only.

A summary of the orientation of N3.35 during the simulations is reported in Fig 6D. In four receptors, AT1h, AT1c, AT1e, and AT2e, we did not observe outward rotamerization of N3.35. N3.35 remained oriented inward, mainly in the *trans* conformation. A high propensity of N3.35 for the outward orientation was observed only in AT2h and OPRD. In addition, an outward rotamerisation was observed in (1) one out of five trajectories of the AT1m variant after 150 ns and (2) one out of three trajectories of the AT1em variant after 220 ns. The *g+* conformation, where N3.35 points towards W6.48, was transiently observed in several trajectories, but, in the presence of sodium, was stable only in the AT2e receptor (S7 Fig). Finally, the simulations of AT1h in the absence of bound sodium revealed a variety of orientations for N3.35. A stable *g+* orientation was observed in the first replica, an outward *g-* reorientation was observed after 40 ns in the second replica, whereas the third replica presented a stable *trans* conformation (Figs 6D and S7).

In the next step, we analyzed the H-bonding pattern of N3.35 in the inward orientation only, to determine whether the outward rotamerization was a consequence of impaired H-bonds (Fig 6E). Most contributions came from D2.50. However, when a serine was present at position 2.49, it contributed significantly to additional H-bonds. The highest total scores were larger than 100% (on average, the side chain of N3.35 was involved in at least one H-bond). They were obtained for AT1h, AT1c and AT1e that have either the N7.46 or the S2.49 pattern. The N7.46S mutation in AT1h induced a decrease in the H-bonding pattern of N3.35 from a total of 109 ± 12% to a total of 64 ± 42% (for four out of five AT1m trajectories, the total was 52 ± 11%). In AT1e, the S2.49A mutation also induced a strong decrease in the H-bonds involving N3.35 from 110 ± 21% to 62 ± 21%. Finally, in the absence of sodium, there was a collapse in the H-bonds involving N3.35 from 109 ± 12% to 32 ± 2%. This indicates that *both* the sodium ion and N7.46 are necessary to stabilize the inward orientation of N3.35 in the AT1h receptor.

As observed for AT1e, in AT2e, S2.49 was also involved in H-bonds with N3.35 (Fig 6E). However, the results were an average of two different cases. In sub-site 1, N3.35 was strongly involved in H-bond interactions with D2.50 and S2.49 (total occupancy of 95 ± 23%). In sub-site 2 (Fig 5F), the occupancy of these H-bonds was low (23%). In AT2h, the total H-bond occupancy of inward N3.35 averaged at 73 ± 32%, which is similar to AT2e and AT1m but contrasts with OPRD (Fig 6E). This indicates that the strong propensity of N3.35 for the outward orientation in AT2h is not related to the absence of H-bonds in the inward orientation. The presence of a methionine at the neighbour position 3.36 was proposed to destabilize AT2h [20]. To support this assumption, in one of the AT2h trajectories, we did observe a bump between M3.36 and W6.48, just preceding the outward rotamerization of N3.35 (S8 Fig).

To summarize the H-bonding pattern of N3.35 observed in the AT1 and AT2 receptors, the inward orientation of N3.35 was always stabilized by H-bond interactions, although at various extents. Thus, in each of the receptors, the collapse of these H-bonds upon the outward rotamerization of N3.35 should destabilize the receptor structure and favour activation, as observed for CXCR4 [38].

For position 7.46, we aimed at comparing the changes in the H-bonding patterns due to the S7.46N mutation. We thus took into account the entire trajectories. H-bonds between residue N7.46 and N3.35 were marginal in all the simulations, except in one AT1c trajectory. This was also the case for AT1h in the absence of sodium. In this latter case, N7.46 was involved only in H-bonds with D2.50 (30 ± 21%). In the receptors with a serine at position 7.46, this residue was involved in H-bonds with D2.50 (> 90% for AT1m, AT2h and OPRD; > 60% for AT1e, AT1em and AT2e). At least for AT1m, these additional H-bonds do not compensate the detrimental effect of the N7.46S mutation on the H-bonding pattern of N3.35 [34]. A hypothesis for this marginal effect might be maintained H-bonds between S7.46 and D2.50 in the active state, as observed in the 5UNF and 5UNG AT2h structures.

### Stability of the sodium binding site through electrostatic interactions

We computed the electrostatic component of the binding free energy of the sodium ion to the different receptors using Delphi. Electrostatic computations were carried out as described in Methods on representative snapshots from MD simulations (every 8 ns of each trajectory). This procedure allows not only an estimate of the average electrostatic free energy but also of its variability within the allosteric cavity during the simulations.

**Fig 7:**
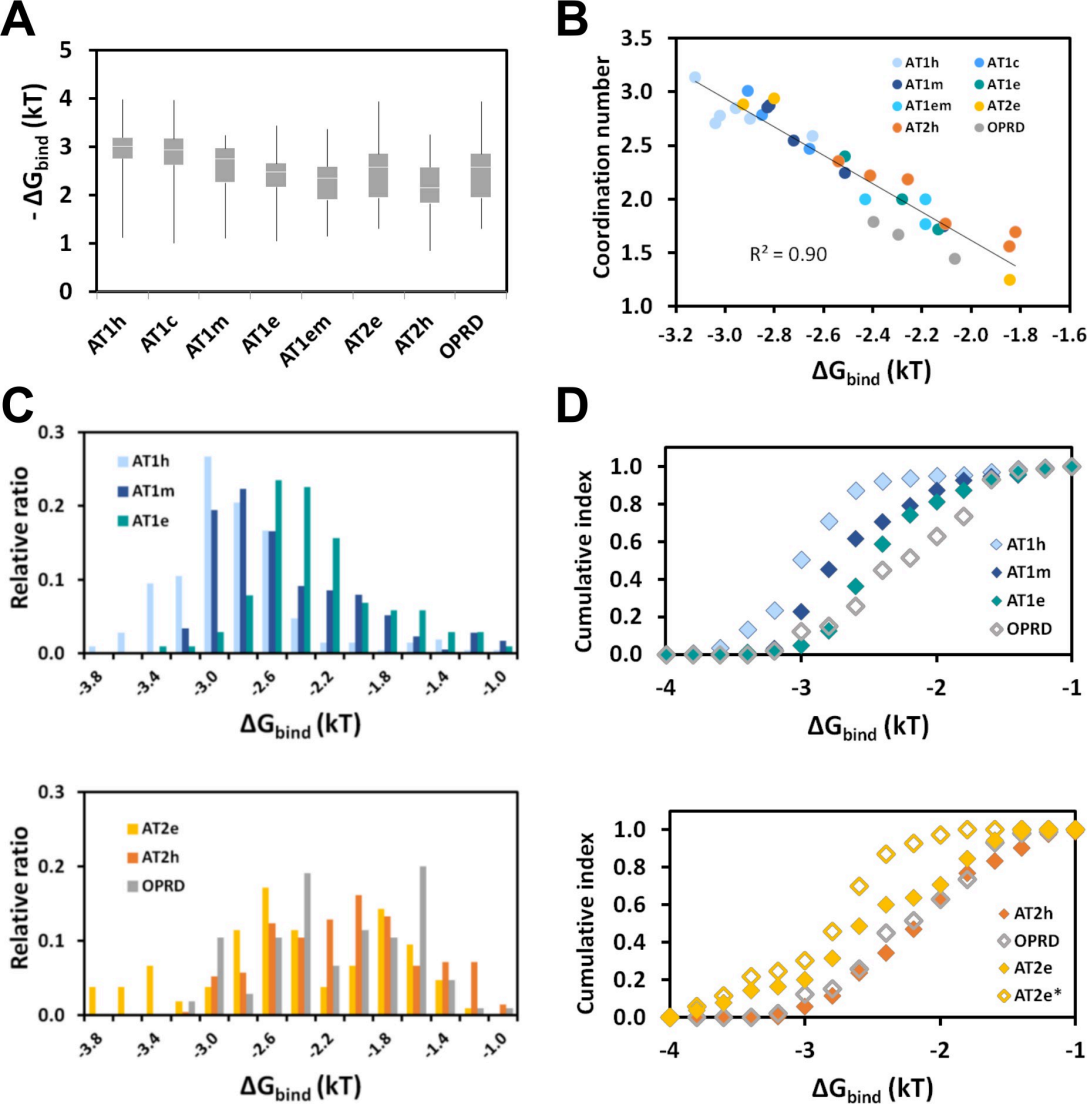
Electrostatic free energy of binding. (A) Boxplots of the electrostatic free energy of sodium binding. The white lines indicate the median. The top and bottom boxes indicate the second and third quartiles, respectively. The winkles indicate the maximal and minimal values; (B) Dependence of the electrostatic free energy of binding on the number of coordination to protein ligands in the first shell. The dots represent individual trajectories; (C) Distributions of the electrostatic free energy of sodium binding to AT1 receptors (top) and to AT2 receptors and OPRD (bottom); (D) Cumulative index of the electrostatic free energy of sodium binding to AT1 (top) and to AT2 receptors (bottom). OPRD (grey diamonds) is shown for comparative purpose. The receptors are indicated by the following color code: AT1h: light blue, AT1c: middle blue, AT1m: dark blue, AT1e: deepteal, AT1em: cyan; AT2e: yellow, AT1h: orange, OPRD: grey. In (A, C, D), the data take into account all the replica of each receptor, except for the open yellow diamonds in (D) corresponding to the high affinity sub-site (replicas 1 and 3) of AT2e (AT2*).

Fig 7A reports boxplots of the electrostatic free energy of sodium binding to the receptors under scrutiny. The medians ranged from -3.0 ± 0.2 kT for AT1h to -2.2 ± 0.3 kT for AT2h but extreme values could reach -1 or -4 kT. The averages were similar to the medians (from 2.9 ± 0.4 for AT1h to 2.2 ± 0.5 for AT2h). For a deeper insight into the variability of the results, for each trajectory, we reported the average free energy of binding as a function of the average number of coordination of the sodium ion to protein ligands in the first shell. As seen in Fig 7B, these parameters were linearly correlated with a coefficient of determination R^2^ of 0.90. We also considered the total number of coordination in both the first and second shells. However, in this latter case, the correlation was modest (R^2^ equal to 0.32).

In most cases, replicas of the same receptors led to similar number of coordination and free energy of binding. The only exception was observed for AT2e. Sub-site 1 displayed high electrostatic free energy of binding (around -3 kT). Sub-site 2 displayed low electrostatic free energy of binding (1.8 kT). These findings reinforced observations based on the N3.35 H-bonding pattern. Nevertheless, the water-mediated H-bonding pattern in sub-site 2 (Fig 5F) was strong enough to maintain this conformation during the 280 ns of the simulations.

The detailed analysis of the distribution of the binding free energy and the resulting cumulative index (Fig 7C and 7D) revealed significant differences between some of the receptors. In particular, for AT1, the electrostatic free energy of sodium binding decreased in the following order: AT1h > AT1m > AT1e > OPRD. For AT2, the electrostatic component of the free energy of sodium binding decreased in the following order: AT2e > AT2h ≈ OPRD. This order was still more marked when only the high affinity site of AT2e was taken into account (AT2e* in Fig 7D).

The electrostatic component is only a part of the total free energy of sodium binding. This one includes components from the reorganization of the receptor and of water, which are exemplified by, respectively, the H-bonding network of N3.35 induced by sodium (Fig 6E) and by the desolvation of the sodium ion observed during the kinetics simulations (Fig 3A). These components should have important entropic contributions. Nevertheless, in spite of inherent limits, the estimate of the electrostatic free energy of sodium binding to AT1 and AT2 yields values that are consistent with OPRD.

## Discussion

Most class A GPCRs possess a highly conserved sodium binding site. The best known effect of sodium is its role as a negative allosteric modulator (NAM), with a strong stabilization of the inactive state. However, the role of the sodium ion is not limited to this “classical” NAM effect. Presence of sodium has dual effects on both reduced basal signaling and increased receptor stimulation by full agonists (reviewed in [26]). Some MD studies observed a transfer of a sodium ion through the membrane during receptor activation, due to the intracellular egress of the sodium upon activation [39]. Coupling of the sodium transfer from the extracellular to the intracellular side of the membrane is likely to make GPCRs sensitive to the electrostatic potential of the membrane [40,41]. Finally, mutations at the sodium binding site can induce biased or impaired signaling, suggesting a role as an allosteric cofactor of class A GPCR signaling [26].

In view of the putative sodium functions and of the role of residues within the sodium binding site of AT1 in the allosteric control of biased signaling [16,23], the question of sodium binding to the AT1 receptor is of primary importance to understand the activation mechanism of this receptor. To answer this question, we first deciphered the evolutionary history of the AT1 and AT2 receptors, with special focus on the sodium binding site (Fig 1). While the sodium site in AT2 was maintained through evolution with few exceptions (Fig 1), the sodium site of AT1 underwent an unusual S7.46N mutation during the transition to the terrestrial life, suggesting that sodium binding might be impaired [16].

Arguments against maintained sodium binding to human AT1 rely on the absence of significant negative allosteric effects of sodium on receptor basal activity [34] and agonist affinity [16,18]. However, as detailed in a recent review [26], classical NAM effects on agonist binding have not been observed for at least one receptor (the β1 adrenergic receptor) whose sodium-bound structure has been resolved. Reversely, absence of sodium in crystallographic structures of putative sodium binding receptors is frequent.

Arguments supporting maintained sodium binding to human AT1 rely on several lines of evidence:

1. The N7.46S AT1h variant displays a constitutive activity at low but not at high sodium concentration. Such a behavior is usually interpreted as strong evidence for the stabilization of the inactive state by sodium binding [26].
2. The pKas of the D2.50 residue in AT1 and AT2 receptors from humans and eels are similar to those computed for OPRD (around 4, see Fig 2A). Consequently, for all the receptors under scrutiny, D2.50 should be negatively charged at neutral pH. The sodium cavity is large enough to contain at least four or five water molecules (Figs 5 and 6), indicating the absence of steric hindrance to sodium binding in AT1h.
3. A sodium ion can actually reach its binding site in human AT1 from the extracellular site (Fig 3). The binding kinetics is very slow as compared to OPRD and CXCR4 (Figs 3 and S2), due to the positive potential of strictly conserved K5.42 (Fig 2B). The kinetics should be similarly slow in any AT1 or AT2 receptor, including the N7.46S AT1 variant (S3 Fig). A consequence of the strongly hindered sodium entrance (low on-rate) is that the sodium dissociation (off-rate) should also be very slow, except upon activation which opens an egress pathway towards the intracellular side. This suggests that the apparent lack of sodium effect on AT1h in experimental data might result from kinetics factors.
4. Comparative analysis of the sodium coordination in the different receptors support the hypothesis of an “efficient” sodium binding mode in AT1h, with 3 interactions with protein groups that maintain the ion in the close vicinity of D2.50 (Fig 6). This agrees with the electrostatic free energy of sodium binding around -3 kT for AT1h, significantly higher than the value measured for OPRD, around - 2kT (Fig 7).

Taken together, these results strongly support the assumption that a functional sodium binding site has been maintained in AT1 throughout evolution. This is also the case for AT2 whose sodium binding site is similar to OPRD (Figs 6 and 7). However, the sodium binding modes markedly differ between eels and humans for both AT1 and AT2, in link with hallmark mutations in the evolution of these receptors during the transition to terrestrial life.

The presence of an asparagine at position 7.46 is a unique feature of amniota AT1. Several important consequences result from this pattern. The first consequence can be inferred from the promiscuity of the N7.46S AT1h variant [34]. The N7.46S variant can be activated by Ang1-7, AngIV, and Ang5-8. Quoting Feng, “The promiscuous agonist specificity displayed by N295S […] in this study could be disastrous if a similar mutation were to occur in AT1 receptors in nature” [34]. Actually, in nature, this is the reverse mutation which occurred in amniota (Fig 1B), preventing AT1 induced Gq activation by these peptides.

The second consequence can be inferred from the mechanism of biased signaling of AT1 recently proposed [16,23]. This mechanism relies on the orientation of N3.35 that acts as an allosteric switch between β-arrestin and G protein signaling. Extensive MD simulations have shown that the inward orientation of N3.35 yields an “alternative” conformation that prevents G protein binding, whereas the outward conformation yields the canonical conformation allowing both G protein and β-arrestin binding. Relative stabilities of the alternative and canonical conformations were estimated by replica exchange MD simulations. In classical MD, the conformations were observed sequentially starting from the inward orientation [23]. Thus, the stability of the inward orientation of N3.35 in the inactive state should contribute to efficient β-arrestin-biased signaling. We show in this study that the N7.46 pattern stabilizes the inward conformation of N3.35 in inactive AT1, *in the presence of sodium* (Figs 6D and S7), The stabilizing role of N7.46 is highlighted by comparing the H-bonding pattern in wild type AT1h and in the AT1m and AT1c variants (Fig 6E). This is especially remarkable for the AT1c variant. The H-bonds linking N3.35 to D2.50 maintain the inward orientation in the AT1c variant on the sub-microsecond timescale in spite of the destabilizing M3.36 pattern (Fig 6D and 6E).

The properties of eel AT1 cannot be straightforwardly deduced from the N7.46S AT1h variant. In eel AT1, the inward orientation of N3.35 is stabilized by an H-bonding network linking D2.50, N3.35, S2.49, and T3.38 (Figs 5E and 6E). The unusual S2.49/T3.38 pattern (only three occurrences in human GPCRs) is highly conserved in fish AT1 (Fig 1B), suggesting that it might be important for the structure-function relationship of this receptor. Presently, experimental data on eel or, more generally, on fish AT1 receptors are missing. They would be of great help to better understand the specificities of human AT1.

The sodium binding mode of AT2h presents strong similarities with OPRD (Fig 6). The sodium cavities are almost identical, with a single difference at position 3.43 which is not involved in sodium coordination (Ile and Leu for AT2h and OPRD, respectively), whereas position 3.36 is a methionine. In both cases, we observe a propensity of N3.35 for the outward orientation and a similar sodium binding mode, resulting in similar electrostatic free energy of binding for the sodium ion (Fig 7).

Full characterization of AT2h is difficult in the absence of response in conventional GPCR essays [4,13]. However, the pharmacological profiles based on binding affinities reveal that AT2 lacks the specificity of AT1 for AngII as compared to either synthetic or natural AngII derivatives [22,42,43]. The promiscuous binding of AT2 to AngII derivatives suggests a “relaxed” conformation by analogy with constitutive AT1 variants [22]. The presence of a methionine at position 3.36 has been proposed to facilitate activation of AT2 by AngII derivatives missing the bulky terminal Phe-8 [19]. The outward orientation of N3.35 in the inactive receptor (Fig 6D) prevents the H-bonding network stabilizing the inactive state (Fig 6E) and should contribute to the “relaxed” AT2h conformation.

By contrast with the “relaxed” conformation of human AT2, eel AT2 appears strongly stabilized by sodium binding due to both electrostatic interactions and H-bonding patterns (Figs 6E and 7D). Ang1-7, which can bind to and activate human AT2 [22,42–44], does not activate eel AT2 [45]. These data support the assumption that evolution favoured the promiscuous behaviour of AT2 in amniota.

Further experimental data on fish receptors are mandatory to understanding the physiological consequences of the mutations observed in AT1 and AT2 receptors during the transition to terrestrial life. It would be of primary importance to determine whether fish AT1 is prone to biased signaling. In any case, thanks to the N7.46 pattern, amniota AT1 has been tailored by evolution for efficient β-arrestin-biased signaling. Biased signaling of AT1 was first discovered using *synthetic* ligands [46,47]. The therapeutic potential of biased AT1 ligands was soon acknowledged because β-arrestin signaling is cardioprotective and enhances cardiac performance [48–50]. More generally, development of biased ligands and understanding of underlying mechanisms are an intense research area for drug discovery. Search of *naturally* biased signaling did not receive such attention [51]. However, the evolutionary driven AT1 propensity for bias signaling prompts questions on the underlying endogenous ligand or mechanism. A putative candidate would be Ang1-7. This peptide is an agonist of AT2 and of MAS [44] and a β-arrestin-biased agonist of AT1 [52,53]. Cardioprotective effects of Ang1-7 are in part related to its activity as a biased agonist of AT1 [53]. Another possibility would be mechanotransduction. Indeed, AT1 responds to mechanical stimuli in the absence of agonist [54]. The underlying mechanisms involve β-arrestin signaling [55–57]. Most interestingly, the Frank-Starling law of the heart (increased cardiac filling leads to enhanced contractibility) depends on the β-arrestin-biased activation of AT1 by mechanical factors [58]. This mechanism is of fundamental importance in the physiology of vertebrates [59].

The transition from aqueous to terrestrial environment required dramatic changes in the cardiovascular physiology [60]. Fishes have a single circulatory system with a two chamber heart. Terrestrial life requires uptake of oxygen from air, which led to the emergence of lungs, of two circulatory systems, and of a heart with three (amphibian) or four chambers (amniota). The two circulatory systems have very different blood pressure, in particular for amniota which need high pressure in systemic circulation to fight gravity and low pressure in lungs to favor oxygen exchange [60]. These physiological changes were accompanied by major changes in the renin-angiotensin system, with the emergence of novel receptors such as MAS and the development of the counter-regulatory axis of the RAS. The conventional activation of a G protein by AT2, observed in eels [45], has been lost in mammalians [4,13] whereas novel receptors, such as MAS, emerged in terrestrial animals [61] and participate in the counter-regulatory arm of the RAS [3,44].

The key mutations observed in AT1 and AT2 (Fig 1) accompanied the transition to terrestrial life. They highlight how a few mutations at a strategic site participate in protein functional evolution. Comparative studies on AT1 and AT2 receptors from different species will lead to a deeper understanding of human cardiovascular regulation and physiology.

## Methods

### Sequence retrieval, clustering and analysis

AngII receptor sequences were obtained from Uniprot (release-2018-09) [62]. To avoid misclassification issues, we carried out an initial exhaustive request using the keywords “angiotensin” and “IPR000276” (Interpro identifier for class A GPCRs), with a sequence length ≥ 260. This led to 550 hits. To eliminate false positives or truncated sequences, a multiple sequence alignment was carried out with ClustalX [63], and then a manual selection was carried out using GenDoc [64]. We obtained a set of 318 “clean” sequences that could be classified into 203 AT1 and 115 AT2 sequences based on a preliminary tree.

The over representation of highly similar mammalian sequences in this set (120 mammalian AT1 sequences out of 203, 68 mammalian AT2 sequences out of 115) prompted us to build a non-redundant (NR) set using the perl script NRDB.pl [28]. This program builds clusters with a sequence identity threshold of 90%. This approach led to 37 and 38 clusters from the AT1 and AT2 sequence sets, respectively. Among them, there were 3 and 5 mammalian clusters for AT1 and AT2, respectively. The aligned sequences of each cluster were visually inspected. The selection of the representative sequences was based on the quality of the sequence (full sequences with longest termini were privileged) and of the genome (well-known species from reference genomes were privileged). The alignments of the clusters were used to verify that the sequence patterns observed in the NR sequences were also valid for the whole cluster.

A Neighbour-Joining (NJ) phylogenetic tree of the aligned representative sequences of the clusters was built with the MEGA5 software [65] using 500 replicates for bootstrapping. The logo plots were obtained from the Weblogo site [66].

Noteworthy, there was no sequence of AT2 from cartilaginous fishes reported in Uniprot. To determine whether the absence of AT2 sequences was due to sequencing problems or to a real evolutionary difference, we extended the search of AT2 sequences to Genbank. In the two additional shark genomes reported, we found AT2-like sequences that should be non-functional, either because of an AngII binding impairing K5.42I mutation (*Rhincodon typus*) or of an N-terminal truncation (*Chiloscyllium punctatum*). No other example of such mutations was observed in the entire set of reported AT2 sequences, supporting the assumption that functionnal AT2 gene was lost in cartilagineous fishes.

### Molecular modeling

The inactive state of AT1h with bound sodium was modeled from the sodium-free 4YAY [17] and 4ZUD [18] structures of AT1 and from the sodium-bound structure of OPRD (4N6H) [36], using the homology modeling software MODELLER [67]. The sodium-bound AT1h was modeled from residue 17 to 317 with 7 water molecules in the sodium pocket positioned by homology with 4N6H. The C-terminal helix 8 (H8) has been modeled by homology with 4NH6 because in the 4YAY and 4ZUD structures, H8 is either missing or bent relatively to the membrane plane. The bent orientation of H8 led to swing-saw motion of this helix and of TM7 during MD simulations [68]. The apo model of AT1h without bound sodium was obtained by replacing the sodium ion by a water molecule. The model of the N7.46S AT1h variant was prepared from the model of AT1h using the Charmm-Gui interface [69]. The model of the chimeric AT1c variant was prepared using MODELLER from the model of AT1h by substituting the sequence from residue 3.25 to 3.40 by the equivalent positions in AT2h [35]:

AT1h: CKIASASVSFNLYASV
AT2h: CKVFGSFLTLNMFASI

Two models of inactive human AT2 were built using MODELLER from the active-like 5UNG AT2 structure [21] and from the inactive AT1h model. The intracellular halves of TM5, TM6 and TM7 and the C-terminal H8 from the 5UNG structure were truncated and replaced by the equivalent parts of the AT1h model, including the sodium ion and the water molecules. The two models differed in the length of TM6. In model A, TM6 had the same length as in AT1h. In model B, α-helical restraints were added to the modeling procedure to insure that TM6 length was identical to the length in the 5UNG structure. In either case, the AT2h models were built from residue 35 to 333 and contained a sodium ion and 7 water molecules in the sodium pocket. In addition, N3.35 was modeled in the *trans* orientation as observed in the inactive AT1 structures (PDB 4YAY and 4ZUD) and faced the sodium binding site.

Models of sodium-bound AT1e and AT2e from *Anguilla japonica* were built with MODELLER by homology with the models of AT1h and AT2h (model B). The S2.49A AT1e variant was obtained from the Charmm-Gui interface. For OPRD, we used the 4N6H structure of OPRD with bound sodium and water molecules [36]. A point mutation in this structure was reversed to the wild type sequence with MODELLER.

### Molecular dynamics simulations

The models were prepared for molecular dynamics simulations using the Charmm-Gui interface [69]. The models were embedded into a palmitoyl-oleoyl-phosphatidyl-choline (POPC) bilayer with 60 lipids on each layer, and then solvated with a TIP3P model for water molecules with all atoms represented explicitly. For simulations aimed at analyzing the sodium binding mode in models with a sodium ion in the allosteric site, the charges were neutralized by adding chloride ions. For simulations aimed at analyzing the sodium pathway towards the binding pocket, the sodium ion was replaced by a water molecule and the charges were neutralized by adding either 0.15 M or 1M NaCl. In all the simulations, the D2.50 residue located within the sodium binding site was negatively charged, in agreement with the pKa of this residue that was calculated with DelphiPKa [32].

Molecular dynamics simulations of the receptor models embedded into the hydrated POPC bilayer were carried out using NAMD 2.13 MD software [70] and the Charmm36 parameter set [71,72]. They were performed using the HPC resources of IDRIS, granted by GENCI (www.genci.fr).

The entire assembly was subjected to energy minimization for 5000 steps to remove close contacts between atoms. Equilibration of protein–membrane system was carried out as previously described [27]. The equilibration protocol started with a first 1 ns phase with six steps in which harmonic restraints on protein and lipids were gradually taken off to achieve a smooth relaxation. This phase was followed by visual inspection of the resulting structure, and then by a 20 ns phase carried out under the same conditions as the production run to achieve stable conditions. No restraint was added to the sodium ion. In the first two equilibration steps of the first phase, the NVT ensemble at 310 K and a time step of 1 fs were used. The following equilibration and production steps were carried out at constant temperature (310 K) and pressure (1 atmosphere), using a 2 fs time-step for integration. The Particle Mesh Ewald method (PME) was used to calculate the electrostatic contribution to non-bonded interactions. The cutoff distance for van der Waals interactions was 12 Å with switch starting at 10 Å. The SHAKE algorithm was applied to the system. Each trajectory lasted a total of 280 ns.

For each receptor, the simulations (including the minimization/equilibration steps) were repeated at least three times from the same build (five replicas for AT1m). For AT1h, there were 2 builds with three replicas (runs 1, 2, 3 for build A, runs 4, 5, 6 for build B). For AT2h, there were 2 models/builds with three replicas (runs 1, 2, 3 for model A, runs 4, 5, 6 for model B). The other receptors had a single build.

The accelerated molecular dynamics simulations were based on the biased potential MD approach developed by McCammon and co-workers [73,74]. In this technique, the true potential energy landscape is modified by adding a bias potential that enhances the escape rates from potential wells, yielding accelerated molecular motions and enhanced conformational sampling. We used the dual boost method as implemented in NAMD [75]. This method works by adding two boosts: (1) the dihedral boost [74] is applied to the dihedral energy of the system and accelerates rotamerizations, and (2) the potential boost [73] is applied to the electrostatic and van der Waals energies of the system, including water and ions. Both boosts are necessary for efficient sampling of protein conformational space [76]. Boosts are applied only when the corresponding energies are below a threshold [73,74]. In the “soft” protocol that we use [76], the threshold energies are set to the average energies, *E_dihed_avg_* and *E_pot_avg_*, computed from classical MD simulations and the acceleration factors are calculated according to:

$$\alpha_{\mathrm{dihed}}=\lambda \times \frac{E_{\mathrm{dihed}\_avg}}{5}$$ (1)

$$\alpha_{pot}=\lambda \times N$$ (2)

where the acceleration parameter λ is set at 0.3 and *N* represents the number of atoms in the system. The 20–120 ns range of classical MD simulations was used to calculate the acceleration parameters. The snapshot obtained after 120 ns was used to initiate the accelerated simulation.

Trajectories were graphically analyzed with VMD [77] and PYMOL (DeLano Scientific LLC, San Franscisco, USA). Quantitative analyses were carried out with home-developed scripts, written in tcl, for use with the VMD software, and in R using functions from the Bio3D R package [78]. The cut-offs for sodium coordination were set as follows: (1) For water, 3 Å and 6 Å for the first and second shells, respectively; (2) For protein ligands, 2.8 Å for the first shell and 6 Å for the second shell with the additional requirement of a first shell water molecule within 3 Å. H-bonds were measured with the H-bond facility of VMD with cutoffs of 30° and 3.5Å. For H-bonds involving N3.35, the trajectories with outward rotamerization of N3.35 within the first 50 ns were removed from the analysis. For the other trajectories, only the snapshots with inward orientation of N3.35 were taken into account. Heatmaps were prepared with R using the ComplexHeatmap package [79]. Volumetric maps were computed with VMD. PYMOL was used for figure preparation.

### Electrostatics potential and pKa computations

Electrostatics potentials were computed by a Poisson-Boltzmann approach with the Delphi program [80], using the smooth Gaussian dielectric function developed by Alexov and al [30]. Similarly, the pKa values of the receptor ionizable residues were computed with DelphiPKa using the same Gaussian dielectric function [31,32]. Both Delphi and DelphiPKa have been installed locally. In the Gaussian representation of atomic density resulting into a smooth dielectric function, two parameters have to be defined: the width of the Gaussian function, σ, and the internal permittivity of the protein, εin. The default values for external ionizable groups are εin = 8 and σ = 0.7, whereas the default values for internal groups are εin = 4 and σ = 0.9. In these studies, we aimed at determining the electrostatic properties of D2.50, located at the sodium binding pocket, in AT1 and AT2. The sodium pocket is lined by several polar groups, including the aspartic acid D2.50, and is wide enough to contain a sodium ion and several water molecules. To determine adequate parameters, we compared the pKa of D2.50 in human AT1 and OPRD, as a function of a variety of εin and σ values. For each pair, analyses were done on seven equally spaced frames from MD trajectories. All the combinations of εin and σ values led to similar pKa values for D2.50 in OPRD and AT1h (S1 Fig). Most of them led to a pKa value < 6, consistent with deprotonation of D2.50 in physiological conditions for both receptors. This was not the case for the default values for protein hydrophobic interior that led to a value of 7.3 ± 1.0 for the pKa of D2.50 in OPRD, which is not consistent with the observed affinity of the sodium ion for this receptor [26]. By contrast, the default values for ionizable groups on protein surface led to a D2.50 pKa value of 4.1, which supports a negatively charged aspartic acid interacting with the sodium ion. Consequently, the latter set (εin = 8 and σ = 0.7) was used for both electrostatic potentials and pKa calculations. For each receptor investigated, pKa computations were done on a representative set of seven frames from MD trajectories.

The electrostatics potential maps were calculated with the Gaussian-based method on the receptors placed at the center of a cube filled with an aqueous medium, implicitly modeled by a dielectric permittivity εex of 80. The axes of the cube were oriented along the principal axis of the solute. The edge size of the cube, L, depends on the maximal dimension of the solute Lp (Lp = L * 0.7). The space was discretized with 1.8 grid points by angstrom. The permittivity of the receptor, εin, was 8 and the Gaussian width, σ, was 0.7. The ionic strength was set at 0.15 M. The atom charges and sizes used for the calculations were based on the default Charmm parameters provided by Delphi (Charmm22).

The electrostatic component of the free energy of sodium binding to the receptors under scrutiny was computed with Delphi. We used the grid energy difference method [81] and the Gaussian dielelectric function with the same parameters as previously (εin = 8 and σ = 0.7). The ionic strength was 0.15 M. The grid energy difference method requires three Delphi runs, for the sodium-bound receptor, the sodium-free receptor and the sodium ion. The separated elements and the complex must be kept in the same position in the cube used for electrostatics computations. The electrostatic component of the binding free energy was computed from the difference in the total grid energy (electrostatic energy computed at each grid point):

$$\Delta G_{bind}=E_{grid}(receptor‐Na)–E_{grid}(receptor)–E_{grid}(Na)$$ (3)

To obtain representative sets of the sodium position in its site, we extracted snapshots at equidistant intervals (35 snapshots separated by 8 ns) from each trajectory, yielding at least 100 snapshots by receptor. All the snapshots of the sodium-bound receptor were superimposed on the initial model and saved as PDB files, using VMD. The PDB files for the sodium-free receptor and for the sodium ion were obtained from the sodium-bound receptor PDB files with shell scripts. The size grid (233 points) was large enough to insure an occupancy < 70% in each case.

## Supporting information

S1 Fig Comparison of D2.50 pKa in OPRD and AT1. (TIF)

S2 Fig Binding kinetics of the sodium ion to CXCR4 and OPRD. (TIF)

S3 Fig Electrostatics potentials of the internal cavities of AT1, AT2, CXCR4 and OPRD. (TIF)

S4 Fig Stability of the receptors during MD simulations. (TIF)

S5 Fig Comparison of the sodium trajectories during MD simulations. (TIF)

S6 Fig Heatmaps of the distances between the sodium ion and the putative ligands. (TIF)

S7 Fig Heatmaps of the N3.35 chi1 dihedral angle. (TIF)

S8 Fig Outward rotamerization of N3.35 in AT2h. (TIF)
