# Supplementary figures and images for: Evolutionary information helps understand distinctive features of the angiotensin II receptors AT1 and AT2 in amniota
Source: PLoS Comput Biol. 2022 Feb 24;18(2):e1009732. doi: 10.1371/journal.pcbi.1009732 (PMC8870451; doi:10.1371/journal.pcbi.1009732)

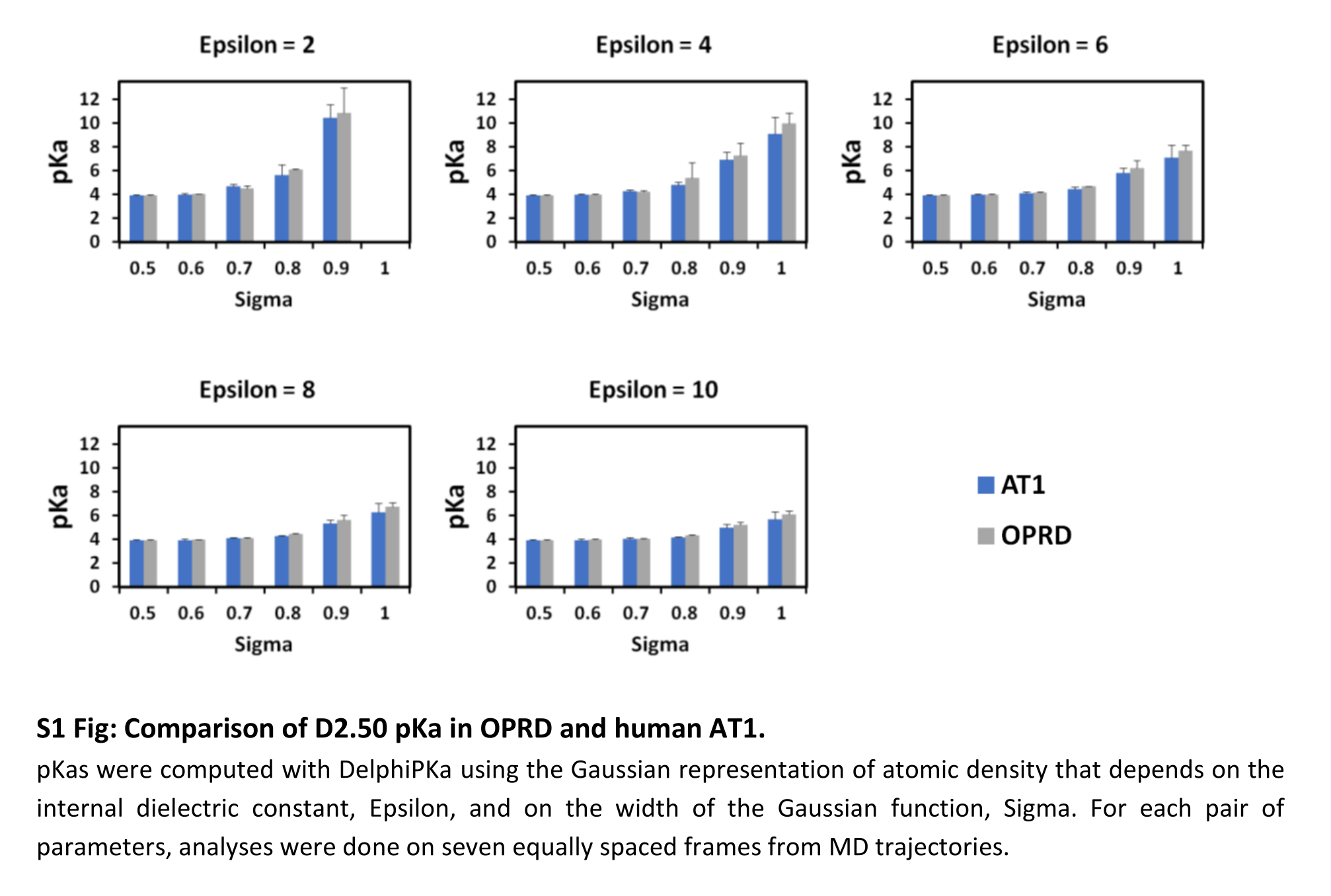

Supplement: S1 Fig — (TIF) [file pcbi.1009732.s001.tif]

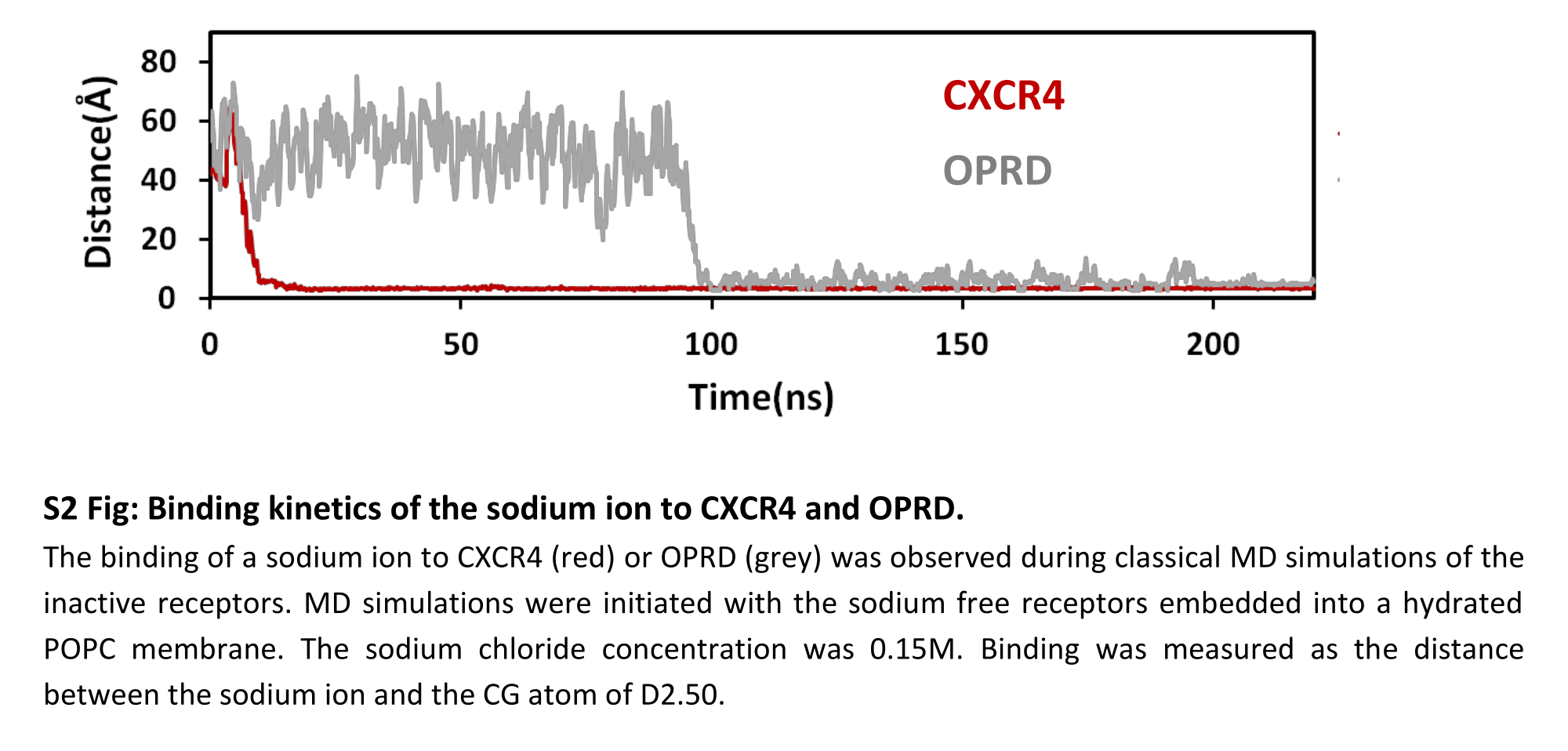

Supplement: S2 Fig — (TIF) [file pcbi.1009732.s002.tif]

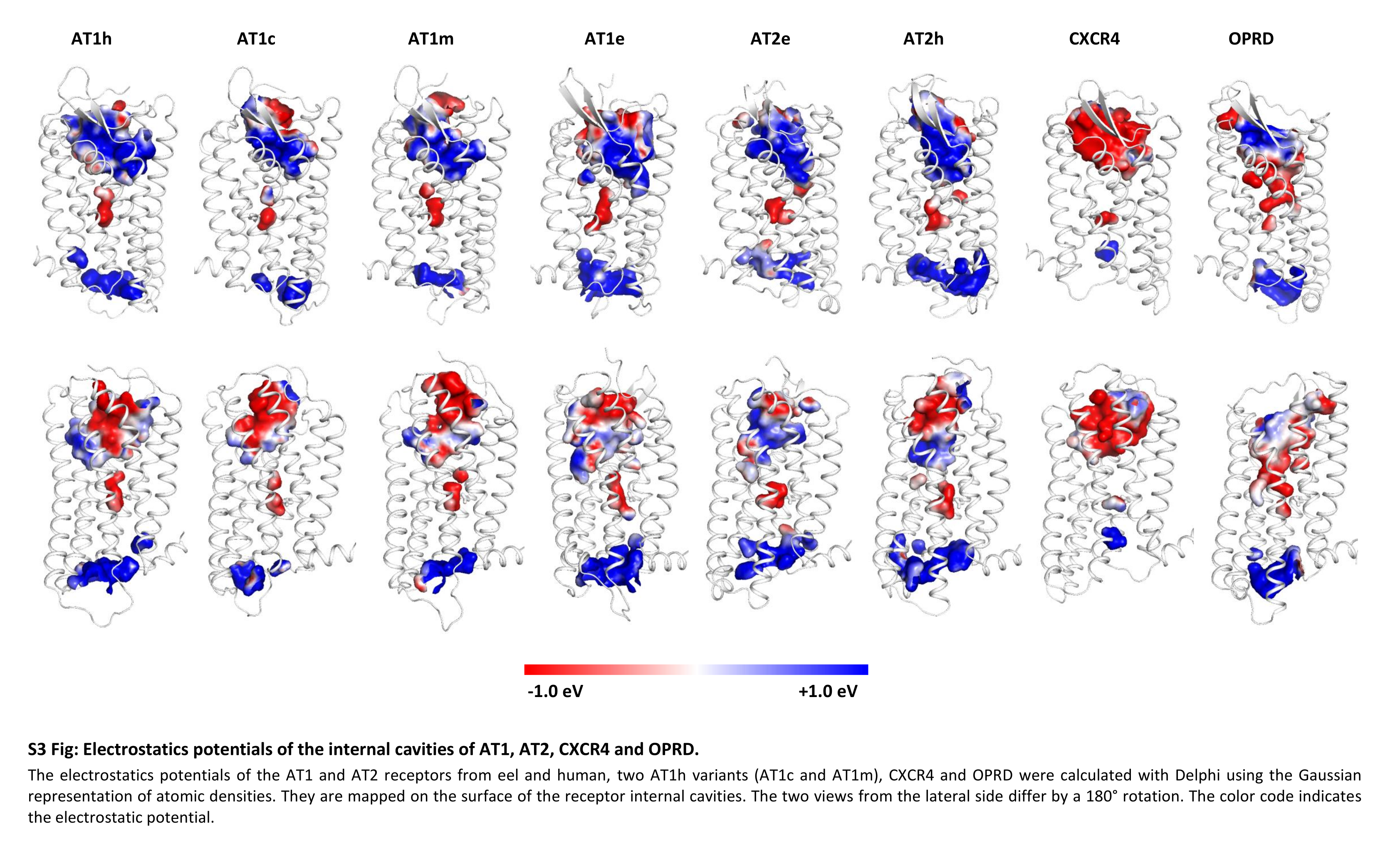

Supplement: S3 Fig — (TIF) [file pcbi.1009732.s003.tif]

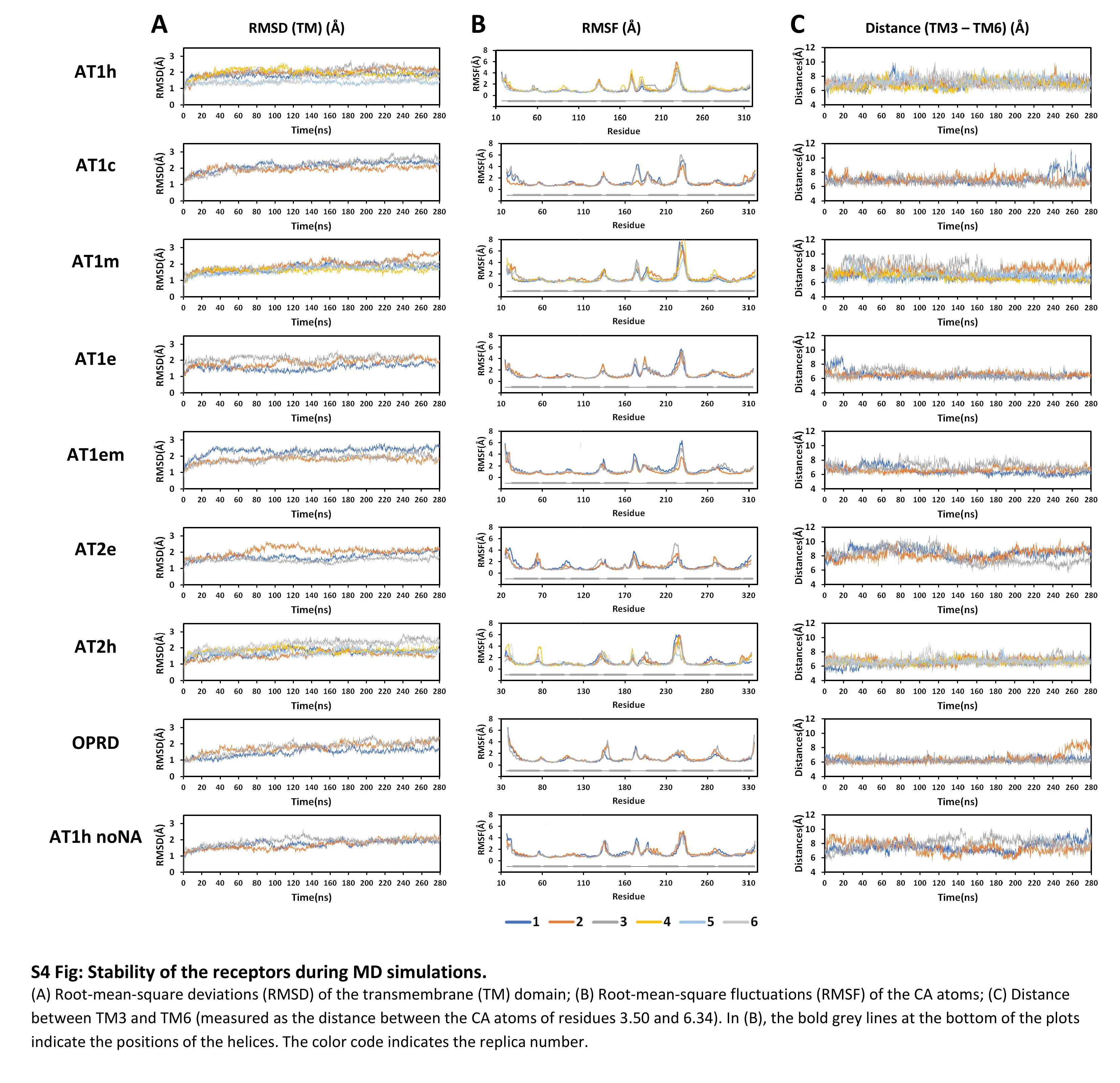

Supplement: S4 Fig — (TIF) [file pcbi.1009732.s004.tif]

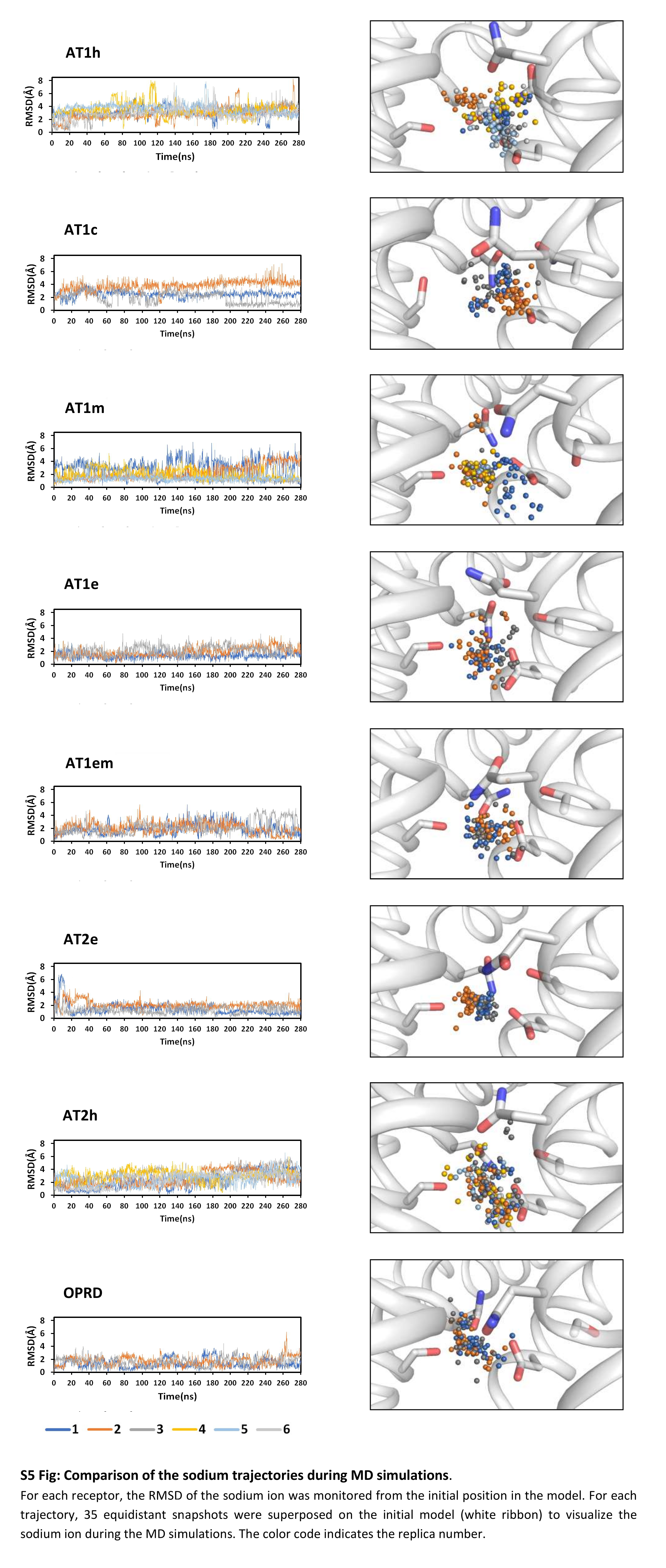

Supplement: S5 Fig — (TIF) [file pcbi.1009732.s005.tif]

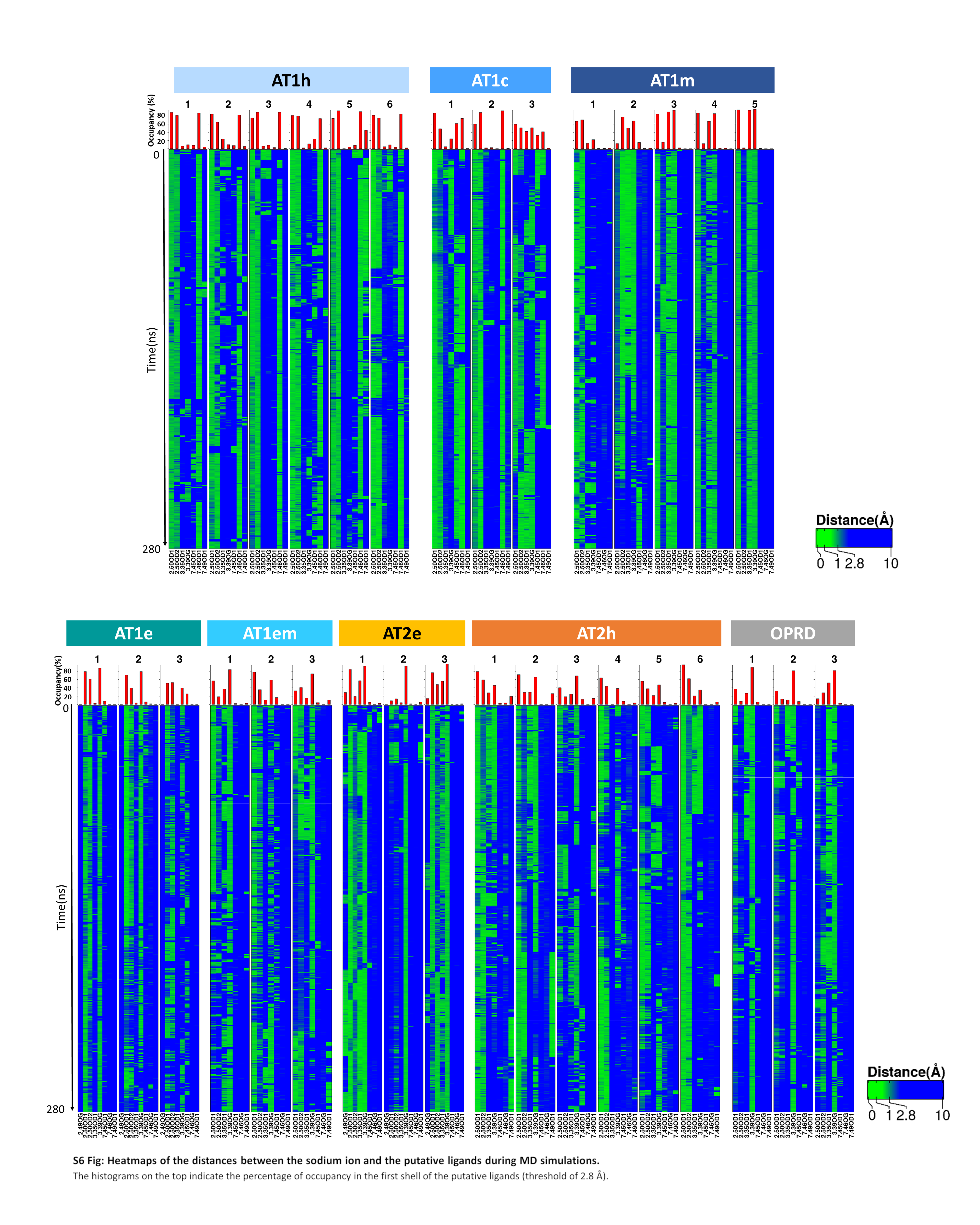

Supplement: S6 Fig — (TIF) [file pcbi.1009732.s006.tif]

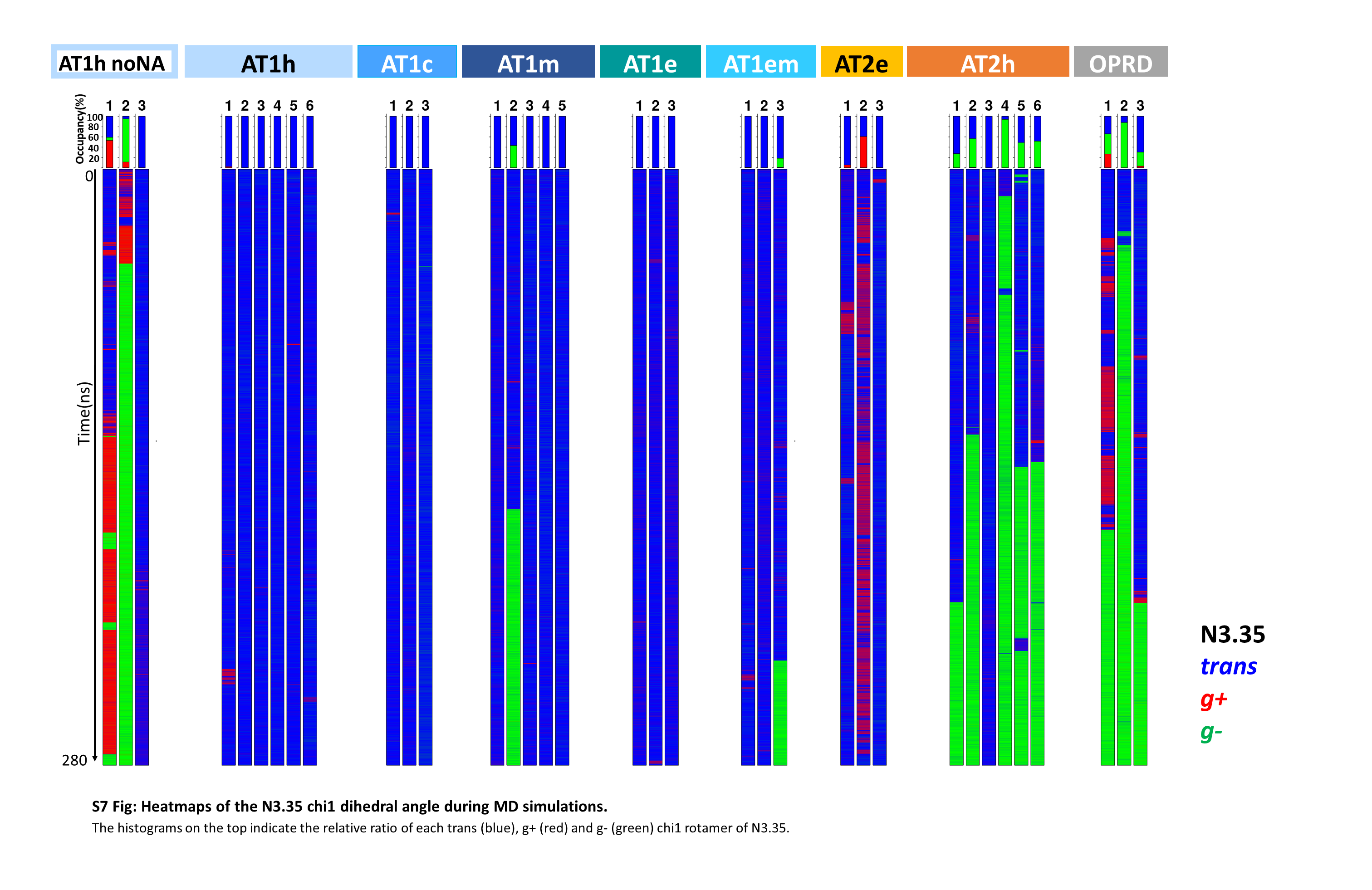

Supplement: S7 Fig — (TIF) [file pcbi.1009732.s007.tif]

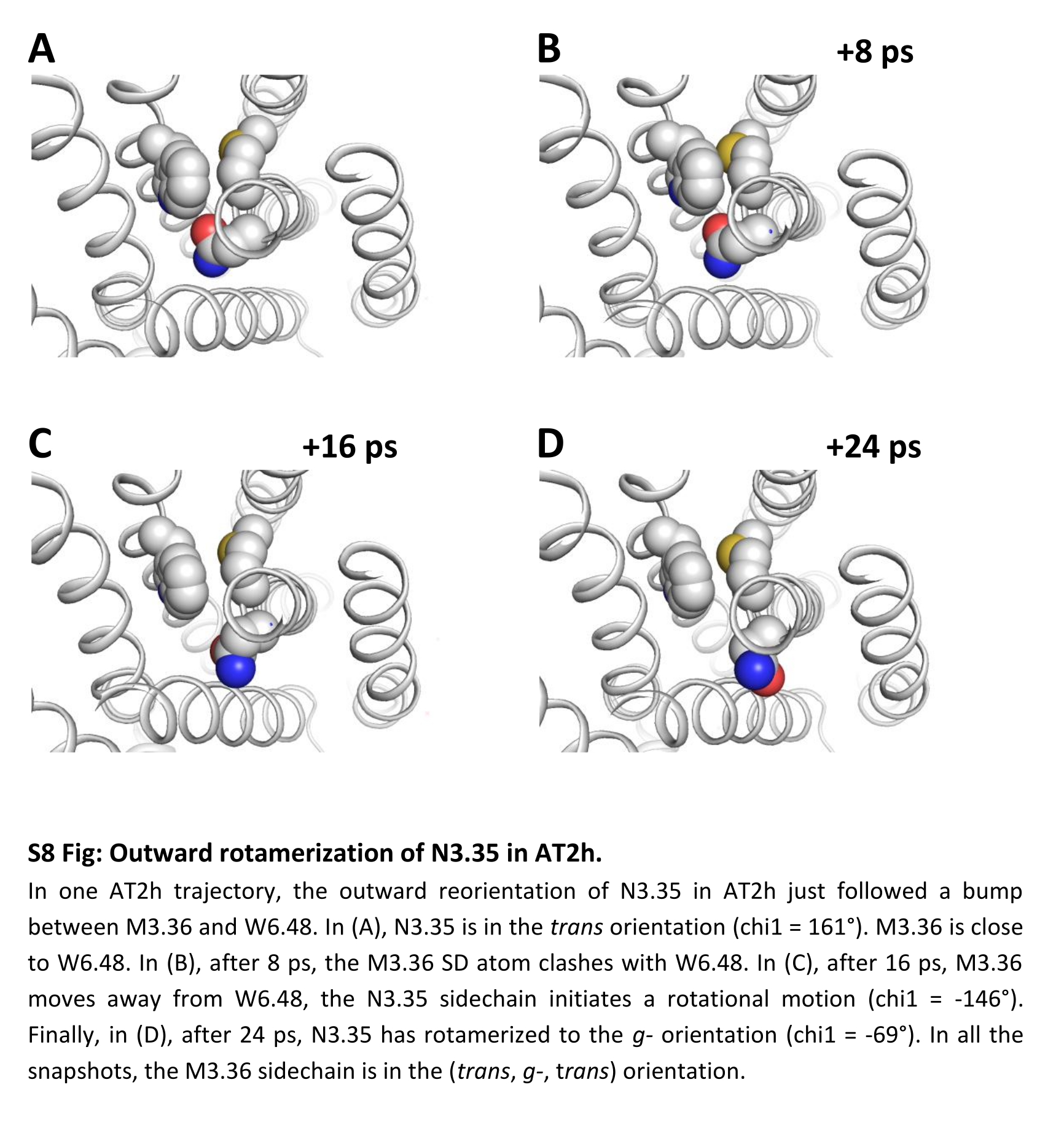

Supplement: S8 Fig — (TIF) [file pcbi.1009732.s008.tif]
